# The Decline and Fall of Materia Medica and the Rise of Pharmacology and Therapeutics in Veterinary Medicine

**DOI:** 10.3389/fvets.2021.777809

**Published:** 2022-01-20

**Authors:** Peter Lees, Wolfgang Bäumer, Pierre-Louis Toutain

**Affiliations:** ^1^The Royal Veterinary College, University of London, London, United Kingdom; ^2^Department of Veterinary Medicine, Institute of Pharmacology and Toxicology, Freie Universität Berlin, Berlin, Germany; ^3^INTHERES, Université de Toulouse, INRA, ENVT, Toulouse, France

**Keywords:** Materia Medica, veterinary medicine, historic, pharmacology, therapeutics

## Abstract

Materia Medica is a Latin term, relating to the history of pharmacy. It describes the sources (vegetable, animal and mineral), nature, preparation, and properties of substances or mixtures of substances, which were used as remedies for the treatment of diseases. Bourgelat authored the first veterinary Materia Medica book. This review describes the evolution and ultimate downfall of Materia Medica concepts and practices. Its survival for more than two millennia reflected the impact of religion and dogmas on therapy. The consignment of Materia Medica to history was signified by publication of the first modern book of veterinary pharmacology and therapeutics by Meyer Jones in 1953. Previously, the dominance of Materia Medica was linked to an hippiatry culture, which was shared with farriers and quacks. The Pasteurian and pharmacological revolutions of the second half of the nineteenth century led to its gradual abandonment. This review explains why the existence of authentically active substances, such as opioid analgesics, cardiotonics and general anesthetics either were not used for those actions or were badly prescribed, in part because of historical precedence and in part from lack of pathophysiological knowledge to justify rational use. The modern concept of dosage, in particular inter-species differences, was not understood. There were also major dogmas, supporting false indications, such as failure to recognize pain as a symptom to be treated, whereas inflammation was only a disease symptom involving excess of activity of the blood system, which had to be vigorously addressed by bleeding and purging. This review covers a well-defined period, ranging from Bourgelat, who wrote the first book of Materia Medica for veterinary studies to the first edition of Meyer Jones textbook in 1953, which marked the end of Materia Medica and the beginning of pharmacology in veterinary medicine.

## Introduction

The discipline of veterinary pharmacology, and its clinical application in veterinary therapeutics, did not devolve from Materia Medica. Rather, pharmacology and therapeutics supplanted Materia Medica and the therapy associated with it. This review describes why, when and how this occurred in veterinary medicine, together with some excursions into the human field. The period covered is 1761–1953.

**Figure 1:**
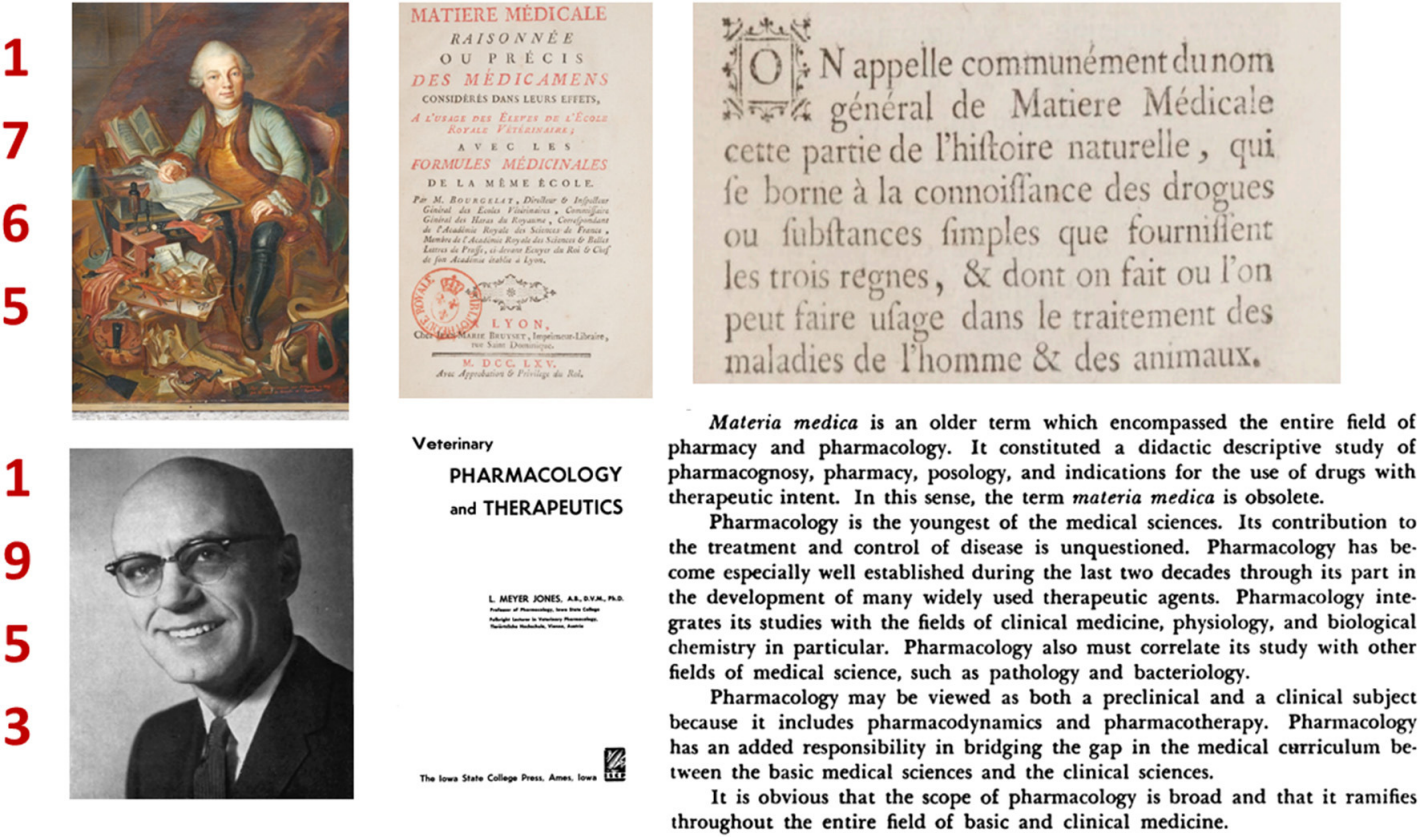
Bourgelat (1) vs. Jones (2): definition of Materia Medica. Definition of Materia Medica by Bourgelat: It is commonly named by the generic name of “Materia Medica,” that part of natural history, which I limit to the knowledge of drugs and simple substances supplied by the three kingdoms, and which are or can be used in the treatment of diseases of man and animal. The three “kingdoms” were animal, vegetable, and mineral. In western pharmacopeias, plants comprised 80% of simples (single substance or products produced by mixing plants) while the remaining 20% was divided equally between substances of animal and mineral origin (3).

In 1761, in Lyon, France, Bourgelat created the world's first modern veterinary school. Four years later, Bourgelat published his book on veterinary Materia Medica (1). Almost two centuries later, in 1953, Meyer Jones (1913–2002), the founding father of veterinary pharmacology and therapeutics in the modern era, published the first edition of his classic textbook “*Veterinary Pharmacology and Therapeutics*” (2). With this, he signed the death warrant of Materia Medica, explaining, “*Materia Medica is an older term which encompassed the entire field of pharmacy and pharmacology. It constituted a didactic descriptive study of pharmacognosy, pharmacy, posology, and indications for the use of drugs with therapeutic intent. In this sense, the term Materia Medica is obsolete”* (Figure 1).

Reviews of the history of veterinary pharmacology from 1950 to 2017 and the uses of antimicrobial agents in animals from 1935 to 2020 have been published (4, 5). After 1975, there was a tsunami of knowledge, leading to the introduction, for veterinary use, of many novel products. This recent history is not reviewed again here. The history of veterinary pharmacology up to 1982 was reviewed by Lloyd Davis in Veterinary Pharmacology and Therapeutics (6). The excellent review of Ruckebusch should also be consulted (7).

## Bourgelat Endorsed for Veterinary Medicine the Dogmas and Precepts of Materia Medica Applied in Human Medicine

Before 1800, the foundational support of medicine by biological sciences was either absent or rudimentary. The writings of the Greek physicians Pedanius Dioscorides (c. 40–90 AD) and Galen (129–201 AD) were medicine's doctrinal bibles. Their Vade Mecum (aide-mémoire) use in human, and later, veterinary medicine, was made with little change up to the beginning of the nineteenth century (3).

Dioscorides wrote a five-volume treatise entitled “*De Materia Medica.”* This was the first pharmacopeia, incorporating accounts of some 600 plants, as well as allegedly therapeutically useful animal and mineral products with almost 5,000 medicinal uses. This seminal work, translated by Beck in 2005 (8), provided the basis for western countries' Materia Medica through the medieval and early modern periods (3). Galen's influence was also dominant. He contributed to our knowledge of anatomy and physiology. However, his doctrinal vision of medicine remained that of Hippocrates (c. 460–c. 370 BC) comprising the theory of the four humors: black bile, yellow bile, blood, and phlegm. It is in this conceptual framework of humorism that, over the centuries, clinicians continued to believe that disease stemmed from an imbalance (dyscrasia) of the body's humors. The optimal approach to addressing these imbalances was to promote the discharge of the damaged fluids, expelling putative morbific excreta. Similarly, to cure inflammatory and other diseases, the aim was to stimulate fluid evacuations from distant organs. The approach was based on the visual observation of critical discharges, which seemed often to resolve the disease (9). This belief accounts for the pivotal role of blood-letting and the use of purgatives, emetics, carminatives, diuretics, expectorants, sialagogues, and diaphoretics. Inflammation was regarded as an imbalance, created by an excessive influx of blood into a given tissue, and not related to any specific pathology. Therefore, bleeding (general or local) was the proposed solution. For example, blood-letting in pneumonia would relieve the blood engorged lung (10).

It is against this historical background that Claude Bourgelat (1712–1779) authored the first veterinary Materia Medica (1). He largely adopted the content and tenets of traditional pharmacopeias, in particular that of London (The *Londinensis* 1618). He emphasized the invaluable character of the latter document. This pharmacopeia (now the *British Pharmacopeia)* was translated from Latin to English by Culpeper in 1649 (11) and it became a major source of information in Europe on the medicinal attributes of plants in treating disease. Bourgelat was an Enlightenment encyclopedist. He also copied extensively from the work of his contemporary Baumé (1728–1804) who published a book entitled “Elements of theoretical and practical pharmacy” (12). This book is in tune with the very limited biological science base of that time, but, importantly, it was linked to the principles of chemistry. Chemistry advanced and was viewed at this time as a disruptive science, having the revolutionary goal of offering alternative explanations to divine goodness to account for the effects of drugs. Bourgelat embraced this reflection. Like all enlightenment encyclopedists, he prudently expressed reservations on the role exercised by God.

Before the Age of Enlightenment i.e., the end of the eighteenth century, medicinal substances were selected for actions consistent with the Hippocratic vision of the functioning of the human/animal body. It was also necessary to address the question, what is the origin of the beneficial effects of products obtained from the three kingdoms, plants, animals, and minerals. The answer was divine will. Paracelsus (1493–1541) developed this concept from the earlier concept of Dioscorides on the theory of signatures.

## The Common Thread of All Therapies: The Doctrine of Signatures or the Doctrine of Correspondences

The dominant paradigm in medicine up to the eighteenth century was that the gods or mother nature had provided the means to cure diseases and relieve pain (13). In medieval Europe, the dogma was that God, in his infinite wisdom and great mercy, had provided man with clues on the origins of nature's therapeutic benefits. It was up to these poor human sinners to first identify and then to use them. Paracelsus (1493–1541) promulgated this concept when writing “*nature marks each growth… according to its curative benefit*.” Jakob Böhme (1575–1624), a German shoemaker popularized the doctrine through his treatise, translated in 2006 (14), entitled “The signature of all things” (1621) which gave the doctrine its name. There were earlier translations, e.g., John Ellistone (c. 1625–1652) (15). Böhme suggested that God marked the objects with a sign, or “signature, which could be read,” indicating their purpose (16). The signature could be the color, texture, shape, smell, and even the environment in which the plant grew (13). The plant might resemble parts of the human body, the shape of an animal or indirectly evoke diseases that it can cure. The British botanist William Coles (1626–1662) wrote a seminal book “*The Art of Simpling*” in which plants were methodically described (name, origin…) together with their virtues and signatures (13). He thus supported the anthropocentric concept that God had given to humans all they needed to heal themselves.

This theological rationale is older than Christianity and has been identified in many non-occidental cultures, including traditional Chinese medicine and Native American and African herbal traditions (17). For example, iron therapy was used by Greek physicians against weakness and anemia, based on the sword as the symbol of strength and power (18). Hippocrates, Galen, Pliny the Elder, and others were aware that willow bark could ease pain and reduce fever. This knowledge lay dormant for centuries and was re-discovered in 1763, when Edward Stone (1702–1768) sent to the Royal Society, “*An account of the success of the bark of the willow in the cure of agues*” (agues=fever) (19). He stated that “*the tree delights in moist soil”* and cited the general maxim that “*many natural maladies have remedies that exist not far from their causes”* (20). The willow tree grew in places with high humidity and had very flexible branches, whilst humans residing in the same places might become stiff, crippled with rheumatism. The notion was that the willow produced for itself a softening substance, which could also benefit man by drinking willow bark decoctions. In this rare case, the like cures like concept was actually true.

Centuries-old beliefs and practices have still not been abandoned, despite the rise of science providing the foundation of evidence-based therapeutics. The beliefs are retained by herbalists and, notoriously, in the practice of homeopathy (21, 22).

## The Principles and Practice of Materia Medica Transmitted Historically as Vade Mecum

Prior to the nineteenth century, knowledge dissemination was based on the library with its books. A retrospective analysis of 12 medical books, spanning more than two millennia and devoted to European Materia Medica, indicated that all were based, with remarkably little variation, on the Dioscordian tradition up to the nineteenth century (3). Accompanying these reference books, the guardians of the doctrine, were a multitude of manuals, guides, cookbooks, diaries…, written by and designed for use by field prescribers. These comprised collections of recipes, proposed or declined by the authors according to their personal empirical experience or imagination.

**Figure 2:**
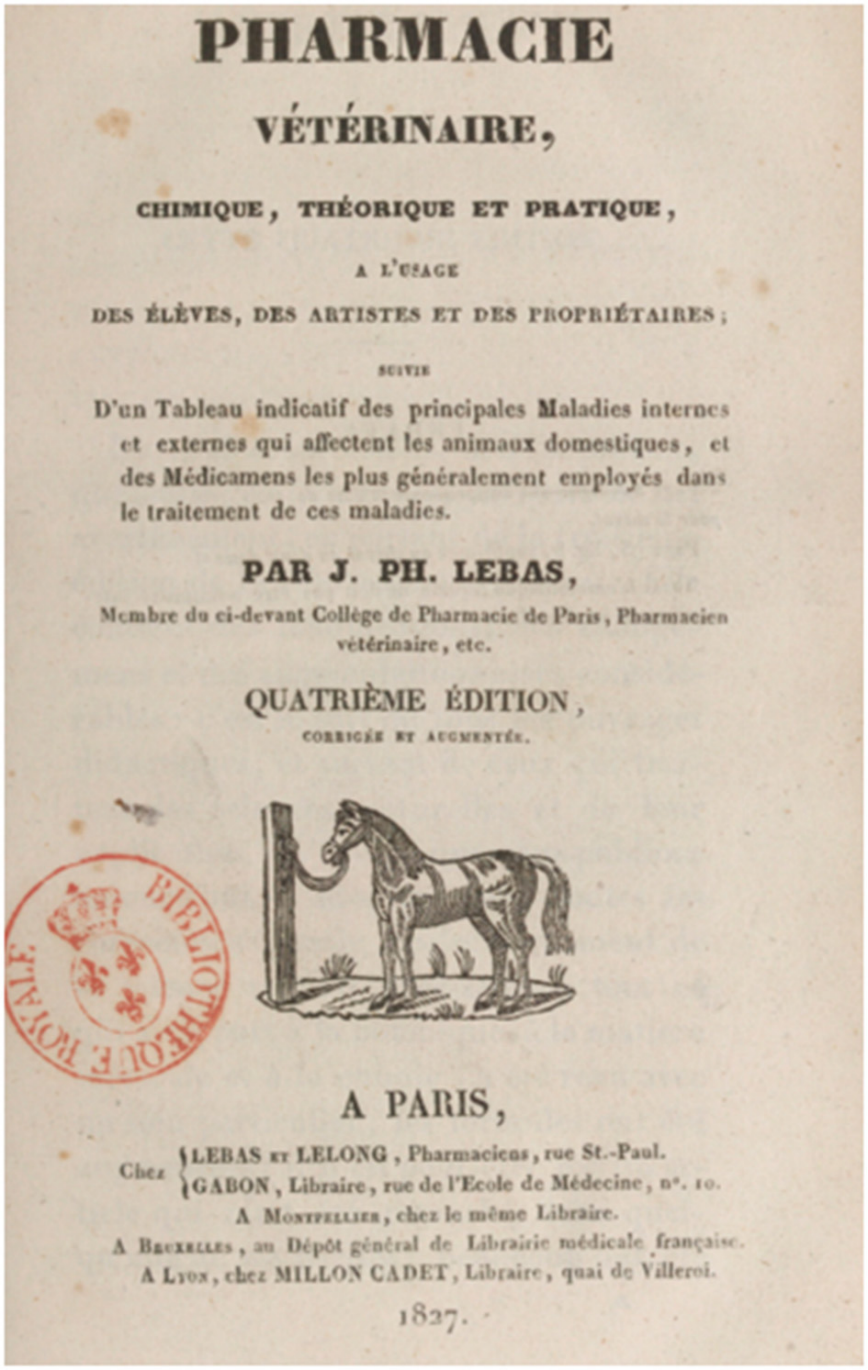
Early French textbook on Veterinary Pharmacy by Lebas (23). Analyzing this 1823 edition (23), Girard (24) one of the most famous professors at Alfort wrote: it is only to aid memory and to facilitate research. The disease being recognized, the artist chooses from the series of medicines which are all suitable for the genre, and many of which might not show up to his memory, the one he considers the cleanest to produce the effect he intends to obtain.

The universal mechanism of action, underpinning all remedies, was based on the goodness of God. Therefore, the books neither possessed nor required any rational presentation of the remedies. Claims for actions and uses required minimal knowledge of physiopathology, which, in any event, did not emerge until the middle of the nineteenth century. Accordingly, the veterinary Materia Medica books were organized like dictionaries, with neither references nor data supporting the claims. The priority was to present the remedies in the most practical way possible, in the form of a *vade mecum* with the simplicity offered by organization alphabetically. For each plant or product, a description was given of source, physical and chemical properties, methods for preparing extracts, methods for formulating them into a wide range of product classes and many modalities of administration and dosage (or rather amount) for each species. Information on the physiological actions on the animal was occasionally attempted (and sometimes fantasized) but, generally, scientific rationale was absent. Most products would have provided no benefit but caused little harm, as the veterinarian's role was that of an artist. In France, successive editions, from 1809 to 1827, of the book by Lebas “*Pharmacie vétérinaire, chimique, théorique et pratique, à l'usage des élèves, des artistes et des propriétaires”* (Veterinary, chemical, theoretical, and practical pharmacy, for the use of students, artists and owners) (23) became the reference text for veterinarians (Figure 2).

## The Emergence of Therapeutics and Veterinary Profession Advances: Qualified and Unqualified Practitioners Including Farriers and Quacks

The development of therapeutics is inseparable from that of the veterinary profession and its education systems. Materia Medica with its recipes, the use of which did not require medical training, enabled a group of non-veterinary personnel, ranging from farriers to simple charlatans, to compete with the evolving veterinary profession. This was possible, as the qualified knew little more than the unqualified (see Supplementary File 1: Veterinarians and farriers). Nevertheless, impacting beneficially and inseparably on the emergence of therapeutics, was the rise of a formally trained veterinary profession, with accredited qualifications.

**Figure 3:**
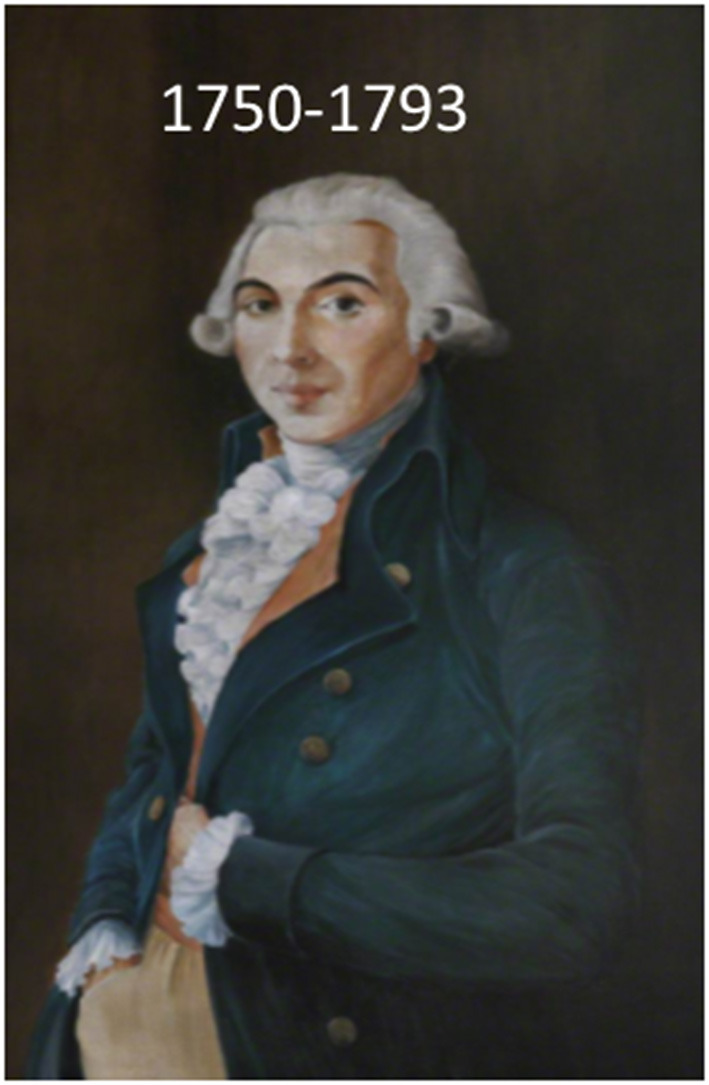
Benoit Vial de St. Bel (1750–1793). Charles Vial de Saint-Bel was born at Lyon (France) in 1753. He studied at the Veterinary School of Lyon, and then was appointed Assistant Professor at Alfort, where he quickly encountered hostility from Bourgelat. On his departure, Bourgelat wrote against Vial's name “Renvoyé comme sujet cabaleur, intrigant et detestable” (Returned as a boastful, intriguing, and detestable subject) and Bourgelat wanted to imprison him in the Bastille. In 1788, he prudently left for England where he married an “English lady of great accomplishments. “He then returned to France before being exiled in 1789 in London at the beginning of the French revolution. There, he met Granville Penn (grandson of William Penn of Pennsylvania) who helped him to express in English his plan to establish a first veterinary school in Great Britain.” On February 18, 1791 “The Veterinary College of London was created; SAINT-BEL was its “Professor.” On August 22, 1793, Charles VIAL de SAINT-BEL, at the age of 40, died of Snot (glanders) (26, 27).

In the UK, a leading figure was the Physician James Clark; his treatise “*A Treatise on the Prevention of Diseases incidental to Horses, from bad Management in Regard to Stables, Food, Water, Air, and Exercise, to which are subjoined Observations on some of the surgical and medical Branches of Farriery, second Edition, corrected and enlarged*.” (25) supported professionalization of the veterinary trade, and the establishment of veterinary colleges. This was achieved in 1791, driven by the campaign of Granville Penn (the founder of the state of Pennsylvania); he persuaded the Frenchman Benoit Vial de St. Bel (1750–1793) (26) (Figure 3) to accept the professorship of the newly established veterinary college in London. In the wake of establishing the first two worldwide veterinary schools of Lyon (1762) and Alfort (1766), there arose many colleges in Europe in the late eighteenth century. The Vienna College (1765) incorporated a hospital (1777) and was the first school of veterinary medicine in German-speaking Europe. Other schools founded were: the Italian college at Torino in 1769, Skara in Sweden in 1772, Leipzig in 1776, Copenhagen in 1777, Hanover in 1778, Dresden in 1780, Budapest in 1786, and Berlin and Munich in 1790. For an overview on the history of veterinary medicine in Germany see (28). Towards the end of the eighteenth century, the Royal Veterinary School of Madrid (1792–1793) was created and modeled on the Alfort school. Its first director was S. Malats, a Field Marshal. Further schools were created at Cordoba and Zaragoza in 1847, Leon in 1852, and at Santiago de Compostela in 1881. As the French model had been followed, the influence of French teaching was considerable. Veterinarians in Spain, as in other schools, were focussed on horses. Malats, who had been trained with Bourgelat at Alfort in France, included in his “Elementos de Veterinaria I” (Elements of Veterinary Medicine I, 1795) a long list of worm purgatives (29).

For Italy, nationhood was not achieved before 1860. Nevertheless, Charles-Emmanuel III, King of Sardinia sent four Cerusici (surgeons) to the Lyon veterinary school in 1764. The small group included Carlo Giovanni Brugnone, who became the first Director of the School and received from Claude Bourgelat a certificate stating his competences “à l'etablissement d'une Ecole semblable à celles ou il a reçu ses intructions.” He is regarded as the father of Italian Veterinary Medicine. The Empress of the Austrian Empire also sent three students to study at the Lyon school in 1772. The Turin school, established in 1769, was run by the Ministry of War along military lines; in 1834 it transferred to Fossano and then to the historical seat of Turin (1859–1999). After 1859, the Royal School of Veterinary Medicine was run by the Ministry of Education.

In Europe, the veterinary schools established in the late eighteenth and early nineteenth centuries arose from political decisions, motivated by military or public health considerations. There were no similar veterinary needs during the colonial periods in the USA. It was not until the war of independence that epizootic diseases arose. Following Philadelphia in 1852, the first veterinary schools were privately run until about 1875, since veterinary education was ignored by governmental agencies (30). The 26 other USA schools were also private until the early twentieth century. Most were non-viable, with few students and operational periods lasted on average about 15 years (31). Consequently, USA participation in academic (32) and scientific veterinary debates was limited. Maurer (33) described this period as the dark age of veterinary medicine in USA. Iowa State College, Division of Veterinary Medicine, founded in 1879, is the oldest and was the first state supported college to inaugurate a 4 year course in 1903 (30). L. Meyer Jones, the father of modern veterinary pharmacology, graduated from Iowa in 1939.

The creation of separate schools or veterinary departments within universities did not suffice to establish the profession. These goals had to await the scientific advances of the nineteenth century, in particular the Pasteurian revolution, which created for the profession the opportunity to establish its pre-eminence over all other competing practitioners of the veterinary art.

## Progress in Chemistry Enabled the Emergence of New Paradigms to Explain Drug Effects and the Synthesis of New Substances

Lavoisier (1743–1794) was the founding father of chemistry and early in the nineteenth century, the discipline was well-established. Many man-made products (acids, alcohols, salts…) were promoted as drugs but without, however, either pharmacological or therapeutic rationale. More importantly, with advances in chemistry, reference to religious authority was no longer so necessary. Rather, the chemical properties of the drugs were harnessed to explain their effects in the new paradigm.

Metals and their corresponding salts were introduced mainly in the nineteenth century. However, the beneficial effects claimed for treatment of diseases were almost always spurious. Taking the example of just one metal, potassium, with its alkali, caustic potash and salts (carbonate, bicarbonate, sulfate, bisulphate, iodide, bromide, nitrate, chlorate, permanganate, and tartrate) each had a range of claimed recommendations, including antacid, antidote to snake bites, rheumatism, skin diseases, pneumonia, eczema, reduced cardiac action, diuretic, sialagogue, destruction of bacteria. The latter action of potassium permanganate, in destroying the spores of anthrax, was reported by Koch (34).

It was during the Enlightenment in 1772 that Priestley (1733–1804), an English chemist, discovered nitrous oxide (N_2_O). In 1799, Davy (1778–1829), also an English chemist, described its euphoric and analgesic effects. He even suggested the possible use of nitrous oxide as a remedy for operative pain (35). His proposal was not pursued and surgeons neglected nitrous oxide for several decades. In 1844, Horace Wells (1815–1848), an American dentist, self-tested nitrous oxide for a dental extraction. Subsequently, diethyl ether was regarded as superior to nitrous oxide as a general anesthetic. Ether was actually known as the “sweet oil of vitriol” in the middle ages and Paracelsus reported its analgesic effects in the dog (36). William Morton (1819–1868) instituted the era of general anesthesia with ether in 1846. Morton had been trained by Wells as a dentist and, after joining the Harvard Medical School, he studied a range of gases. His revolutionary idea was to demonstrate by gas inhalation that the “seat of pain” was in the brain and not at the peripheral site of injury, as then generally accepted (37).

Ether was immediately tested in dogs and cats and results reported in the Veterinarian (38), the reference British journal at that time. However, ether was rapidly replaced by chloroform, discovered in 1847 and claimed to be more potent. Chloroform became popular after its administration to Queen Victoria for the birth of her son, the future Edward the Seventh and the practice was known as “narcose à la reine.” For veterinary medicine (in practice for horses), the routine use of volatile liquids was impractical, in consequence of major difficulties in administration and also the health hazard for both operators and patients. It was even suggested that the anesthesia was largely due to asphyxiation and this was probably, at least partially, true (2). In Germany, both Hertwig (39) and Hering (40) described methods for general anesthesia in horses They suggested soaking a sponge with ether and placing it in one nostril, closed, with the other open to control the oxygen supply, or alternatively placing the sponge in a feed-bag. Both authors mention excitation as only a transient problem.

Chloral hydrate was discovered in 1831 by von Liebig. For veterinary medicine, chloral hydrate, injected intravenously, had a decisive advantage over ether and chloroform. In 1872, Pierre-Cyprien Oré (41) from Bordeaux, administered it first in dogs and then in man. In 1875, Humbert described the effect of chloral hydrate in horses for dose ranging from 30 to 70 g (42). Chloral hydrate was not an ideal anesthetic. The analgesic action was limited and intravenous administration in a large volume was complicated by the risk of phlebitis. Only when barbiturates were introduced did general anesthesia became routine in the dog and cat. With rapid onset of action, they could be used to induce anesthesia, following which intubation greatly facilitated maintenance with inhalational anesthetics in all species. Thiopentone was first described for veterinary use by Wright in 1937 (43) and, over the next 60 years, it was the most widely used induction agent, especially for dogs and cats.

A property of general anesthesia, unlike most Materia Medica products, was their visually obvious effects. However, they were not adopted for routine use in the nineteenth century by horse surgeons, who preferred rapid surgery to general anesthesia. The status of pain in animals was not well-defined, whilst rapid surgery performed by manly men with great manual skill was preferred. Writing in the Veterinarian in 1847, Mayhew, the first to report the use of ether in dogs and cats, indicated concern on observing the excitatory induction stage. He questioned its use compared with rapid surgery. He stated (38): “*We cannot tell whether the cries emitted are evidence of pain or not; but they are suggestive of agony to the listener, and, without testimony to the contrary, must be regarded as indicative of suffering. The process, therefore, is not calculated to attain the object for which in veterinary practice it would be most generally employed, namely, to relieve the owner from the impression that his animal was subjected to torture. There has been yet no experiment that know of been made to ascertain the action of the vapour on the horse; but I cannot anticipate that it will be found of much service to that animal*.” With such assessments, it was never likely that ether would be readily adopted and Percival, the influential editor of the Veterinarian, urged veterinarians “*to lose no time in bestirring themselves to ascertain what may or may not be done, so far as their patients are concerned, by the ethereal stifler of pain and sensibility…” It is now notorious enough that both men and horses have succumbed, under the influence of ether, to rise no more, and, as post-mortem examinations of their bodies has shewn, in consequence of such influence* (38). In contrast, Hering in Germany was enthusiastic for both chloroform and ether in his text “Arzneimittellehre” 1855: Chloroform recently introduced “*is used as a pain reducing narcotic, often in vapor form…. It causes in animals within a short time (few minutes) such numbness that one can perform operations on the animals as on cadavers*.”

## Materia Medica Books in the First Half of the Nineteenth Century Began to Incorporate Scientific Data Which Would Lead to the Rise of Pharmacology and Therapeutics in the Next Century

**Figure 4:**
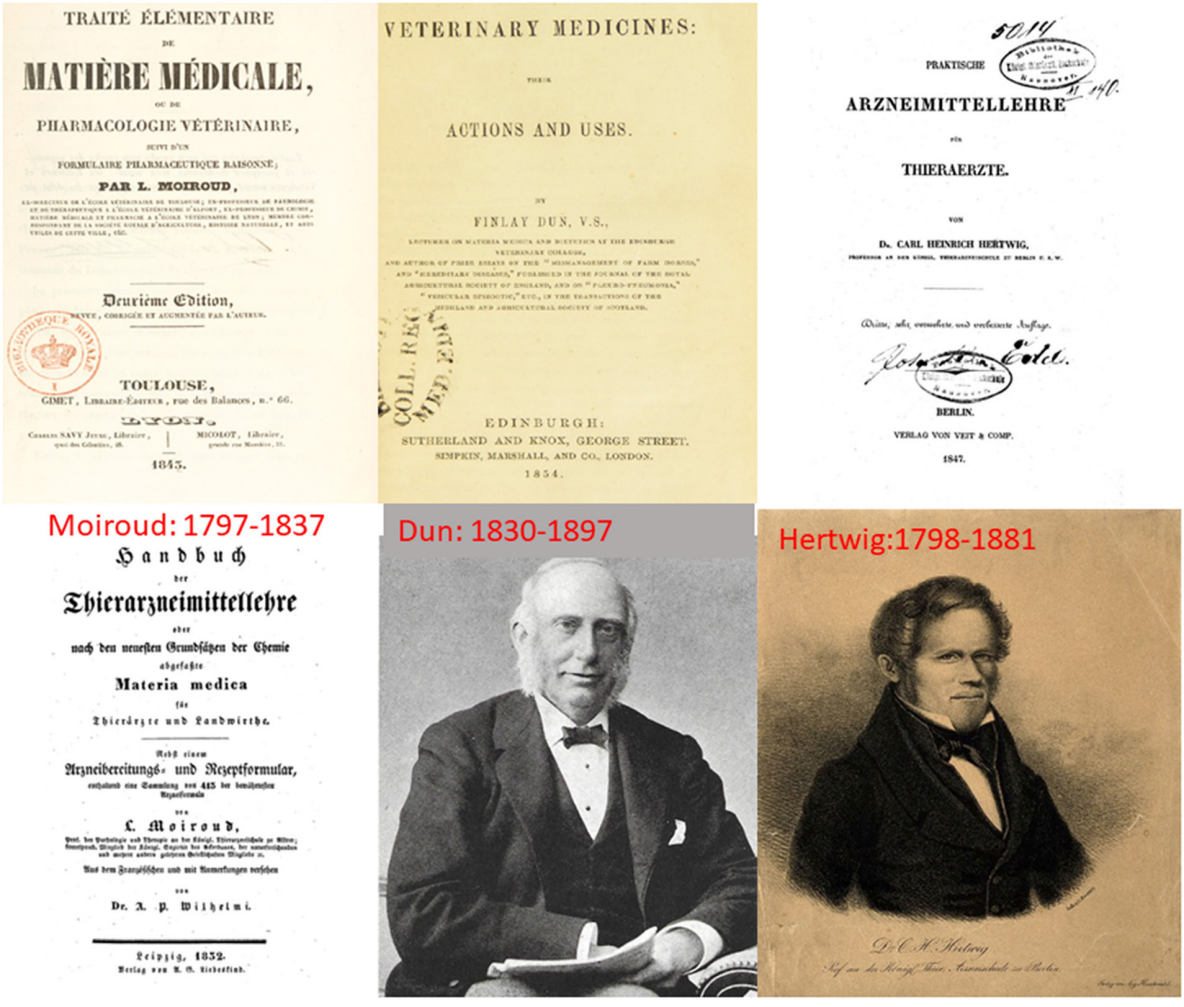
The first three major textbooks of veterinary Materia Medica were published in French, German and English in the mid-nineteenth century. Louis Moiroud (no known picture) was born in 1797. He studied veterinary medicine in Lyon. He became a professor in Lyon in 1824, was appointed to Alfort in 1829 and finally became the Dean of the new veterinary school in Toulouse in 1832. Moiroud published relatively little (he died aged 40) but his book entitled “*Traité élémentaire de matière médicale ou de pharmacologie vétérinaire*” (first edition in 1831) became a standard text in France. This book was immediately translated into German in 1832 (Handbuch der Thierarzneimittellehre, oder nach den neuesten Grundsätzen der Chemie abgefaßte Materia medica für Thierärzte Und Landwirthe. Nebst einem Arzneibereitungs-u. Rezeptformular enthaltend eine Sammlung von 413 Rezepten der bewährtesten Arzneiformeln). Finlay Dun, in the preface to the first edition of his ‘Veterinary Medicine’ in 1853 indicated that the Moiroud book was consulted as well as Hertwig's “Handbuch der Praktische Arzneimittellehre für Thieraerzte.” Carl Heinrich Hertwig was born in 1798. He studied both medicine and veterinary medicine. Hertwig was appointed as a repetitor at the Berliner Tierarzneischule and became head of the clinic for small animals. He had a reputation as a highly skilled surgeon and was appointed as professor in 1833, teaching several subjects including practical pharmacology. Together with Ernst Friedrich Gurlt, he founded the “Magazin für die gesammte Thierheilkunde,” (Magazine for the entirety of veterinary science) in 1835, and this publication continued until 1874. The first edition of his Handbuch der Praktischen Arzneimittellehre für Tierärzte (Practical pharmacology for veterinarians) was published in 1833 (46). He claimed, in the foreword of the first edition, to have performed more than 1,500 practical experiments on animals to evaluate drug effects. Due to its great popularity 5 editions followed, up to 1872 (46). Finlay Dun (1830–1897) graduated from the Royal (Dick) School of Veterinary Studies. He was appointed Lecturer in Materia Medica and Dietetics at the Edinburgh Veterinary College. It is thought that he applied unsuccessfully for the University's Chair in Agriculture in the 1880s. He published in 1854 the first edition of his book “*Veterinary medicine; their actions and uses”* (47). This was followed by 11 editions up to 1913, later editions being revised by other authors whilst retaining his name. These 11 editions reflect the steady advances made in veterinary pharmacology and therapeutics and the slow but sure recognition of the limitations of Materia Medica.

Three textbooks of veterinary Materia Medica, in French, German, and English, dominated the European scene around the mid-nineteenth century. From 1831 to 1845, Louis Moiroud (1797–1831), professor of therapeutics at Toulouse and Alfort published several editions of his text “*Traité élémentaire de matière médicale ou de pharmacologie vétérinaire*”(Elementary treatise on medical matters or veterinary pharmacology) (44, 45). In Germany, Carl Heinrich Hertwig (1798–1881), professor at Berlin published the first edition of his *Handbuch der Praktischen Arzneimittellehre für Tierärzte* (Practical pharmacology for veterinarians) in 1833 (46). Several editions followed, up to 1872. Finlay Dun (1830–1897), Lecturer on Materia Medica and Dietetics at the Edinburgh Veterinary College, published in 1854 the first edition of his “*Veterinary medicine; their actions and uses”* (47). This was followed by 11 editions up to 1913. These several texts give testimony to the progress of knowledge (Figure 4).

For Moiroud, the word pharmacology appears in his title and in the preface and he defines and discusses the disciplines of pathology, pharmacology and therapeutics. He adopted the metric system to express drug dosage and indicated that doses are not necessarily proportional only to animal body weight but depend on multiple species-linked factors. Dosage may also be pathology dependent (health vs. disease). Moreover, he challenged the traditional classification of remedies alphabetically. His classification was based on effect achieved; the two major categories were debilitating agents (inhibitors) and stimulants.

Based on the increasing availability of methods of chemical analysis, together with the introduction of drugs in pure form, Moiroud asserted that, for selected drug examples, absorption can occur from the digestive tract and the skin of horses, cattle, and dogs, followed by distribution in blood and finally elimination, predominantly by the kidneys. Moiroud (45) reported that Eduard Hering (1799–1884), of the Stuttgart veterinary college, injected yellow prussiate of potash into the jugular vein of a horse and detected it in the contralateral jugular within 20 s. Moreover, it was exhaled within a few minutes. Such data were revolutionary for the nineteenth century. The implication was clear; a drug can act at a site distant from the administration site. Hence, it is not only the drug's physico-chemical properties or the methods of its compounding that account for action remote from the administration site. In the same inspirational mode, the notion of mediation of drug action through endogenous mechanisms was recognized. Finlay Dun, in the first edition of his book (47), wrote “*The impression, which they (*drugs*) produce on parts with which they are first brought in contact, is transmitted along the nerve to other parts”* and this mode of action is called “*action by sympathy”* (the concept of sympathy here indicates a common nervous influence).

Whatever the mechanism underlying the systemic effect–either drug transported in blood to its target site or an indirect action via the nervous system, these revelations posed a major problem for Materia Medica. If the drug works at a distance from administration site, how can Materia Medica practitioners justify differences between irritants, emollients, demulcents, protectives, adsorbents, astringents, counter-irritants, bitters, carminatives, cathartics, ruminatonics? How to justify their formulation into a wide product range, including decoctum, infusum, liquor, mistura, aqua, emulsum, syrupus, tinctura, spiritus, elixir, vinum, pulveres, suppositorium, oleum, pilula, unguentum, collodium, emplastrum, collodium, bolus, haustus, each meticulously prepared using a range of pharmaceutical processes, trituration, elutriation, levigation, maceration, digestion, lixiviation, percolation, dialysis, scaling, liquefaction? How to explain the effect of drugs administered by the intravenous route? As discussed by Hering and Weiss (40) “*in this way, you get the pure effect of the drug,”* and all that is required is water solubility.

Another aspect can be added to honor the achievements of this period. In his foreword to the first edition (1833) of his Materia Medica book, Hertwig (46) stressed that he commenced writing only after performing no less than 1,500 experiments on the effects of administered drugs, to confirm or reject earlier claims. He stated that for his own treatments, as Director of the hospital of the school of veterinary medicine in Berlin (1830–1840) he regularly needed no more than 30 of the available drugs.

In the last edition (1872) of his text, Hertwig (39) underscored his opinion that pharmacology should be based on experience, in turn based on repetitive observations (39). He emphasized that repetitions on the same animal with the same drug dose are subject to variability. He further recommended monitoring effects on several animals simultaneously, commencing with the lowest effective dose and performing a dose escalation. Following observations on healthy animals, this should followed by observations on animals with specific diseases for which the drugs are indicated. He recommended incorporating sick animals under the same conditions as healthy animals, as well as untreated healthy animals–an early example of controlled studies.

## Nineteenth Century Scientific Revolutions

### Pharmacodynamics

In the mid-late nineteenth century, evolving initially before and subsequently alongside the Pasteurian revolution (section on Bacteriology), a new era of pharmacodynamics commenced, driven by a new breed of physiologists. They adopted the experimental method to elucidate physiological processes and drug modulating mechanisms. Promoting the rise of pharmacology were Magendie (1783–1855) and his student, Claude Bernard (1813–1878) (48). Magendie challenged historical reports on plant extracts by investigating their active ingredients. Morphine was first isolated between 1803 and 1805 by the German pharmacist Friedrich Sertürner (1783–1841). This is thought to be the first isolation of an active ingredient from a plant. Magendie collaborated with leading chemists, notably Joseph Pelletier (1788–1842). Pelletier isolated emetine (1817) and, in collaboration with Joseph Caventou (1795–1877), he also isolated several major alkaloids, including strychnine (1818), brucine and veratrine (1819), cinchonine (1820), quinine (1820), and caffeine (1821). For each compound, Magendie established their physico-chemical properties, effects on both animals and humans, and finally designing standards for therapeutic use. For example, he conducted foundational research on the dose-effect relationship, drug disposition in the body, mechanism of drug action and structure activity relationships. Through these pioneering studies, Magendie initiated the science of experimental pharmacology, as defined and still understood today.

Claude Bernard (1813–1878) was both physiologist and pharmacologist. His major conceptual and methodological contributions provided the basis of textbook accounts still appropriate to this day. His demonstration of the action of curare, extracted from a South American arrow poison, at the motor nerve-voluntary muscle junction, was a landmark achievement. He also predicated the future principles of pharmacokinetics of drugs and poisons, recognizing the importance of factors such as absorption into, distribution within (including binding to blood) and elimination from the body, in determining magnitude and time course of drug action. He further recognized the value of poisons as experimental tools, enabling physiologists to unravel biological mechanisms of organ systems.

Despite these advances, no major applications of pharmacodynamic principles to therapeutics were possible until clinicians had adopted physiopathological mechanistic concepts. For example, fevers were not regarded as symptomatic of specific disorders; rather, fevers themselves were considered to be disease categories.

### Bacteriology

Louis Pasteur (1822–1895) in France and Robert Koch in Germany (1843–1910) laid the foundations of the science of bacteriology. The Pasteurian revolution was crucial in providing the veterinary profession with its scientific foothold and growing prestige. In France, from 1881, a law granted veterinarians a restricted monopoly on treatment of contagious disease (see Supplementary File 2: Veterinary science and institutional conquests). The law and Pasteur's science provided a paradigm shift, through the development of sera and vaccines. These eclipsed all other forms of therapy and especially Materia Medica. In France, veterinarians immediately became zealous apostles of Pasteurian theories. It was with the support of a veterinarian in 1881, at Pouilly-le-Fort, that Pasteur conducted a famous experiment, demonstrating the effectiveness of his vaccine against anthrax. Just one year later, 270,640 sheep and 35,654 cattle were vaccinated (49).

**Figure 5:**
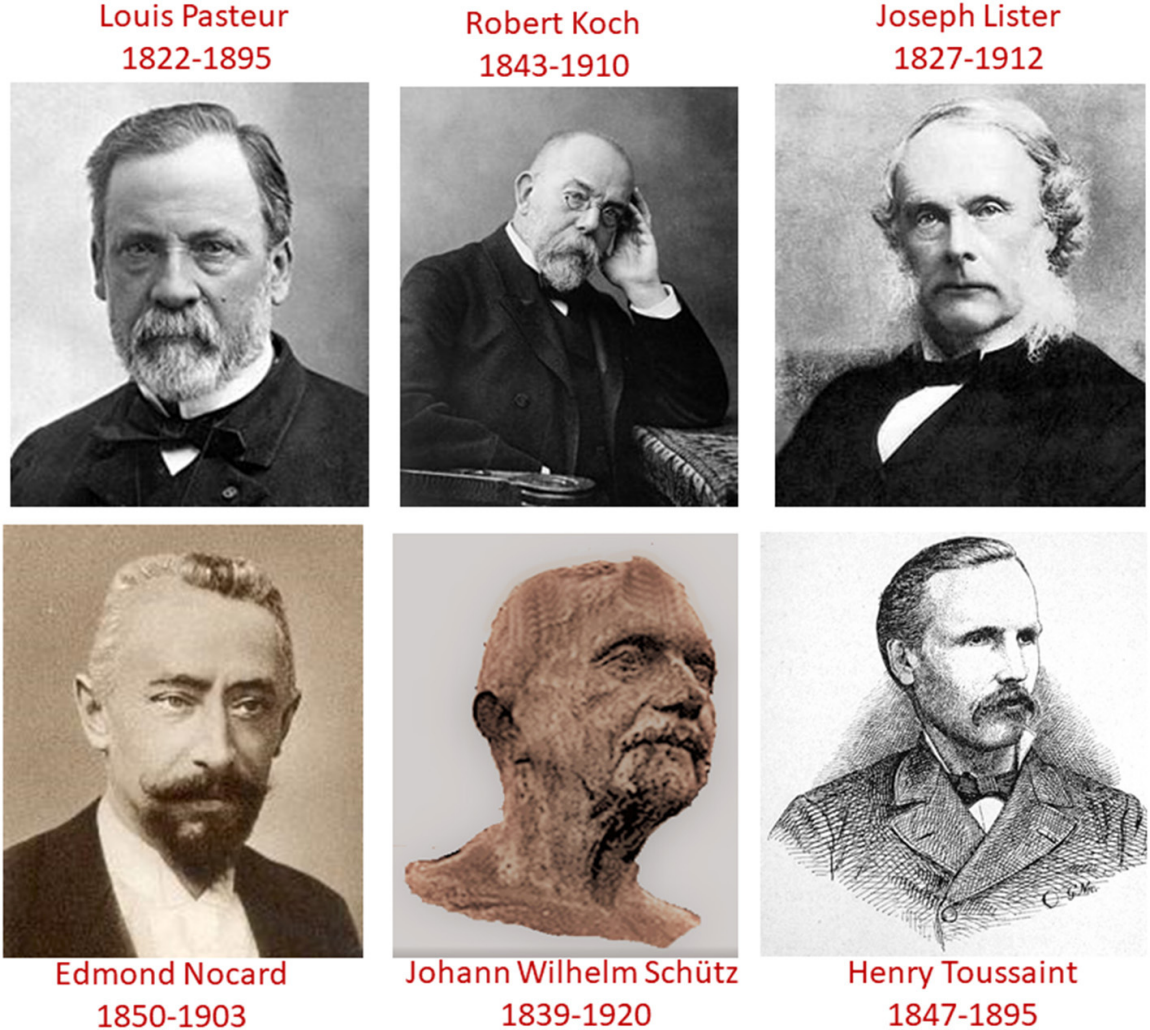
The three early giants of microbiology (Pasteur, Koch, and Lister) and “their associated veterinarians” (Nocard, Schütz, and Toussaint). Pasteur, Koch, and Lister, the three giants, who wrote and researched the history of bacteriology and medical microbiology, had complex inter-personal relationships. This was especially so for Pasteur and Koch with Lister paying the role of mediator. These three greats established friendly professional relationships with veterinarians: notably between Pasteur and Nocard from Alfort; between Lister and Toussaint from Toulouse; and between Koch and Schütz from Berlin. The involvement of veterinarians in this Pasteurian revolution exerted a profound influence on the early history of the veterinary profession; there was immediate adoption of the new scientific paradigms, thus transforming it into a true medical profession, whose activity was henceforth based on science. This was clearly recognized by Pasteur himself during his inaugural speech welcoming in 1884 Bouley, inspector general of veterinary schools in France, as the new president of the French Academy of Sciences with these words: “*A century ago, gentlemen, you had only a modest position in the scientific world… So, gentlemen, while human medicine had confirmed its letters of nobility since the beginning of the civilized world, you were still, in the middle of the 18th century, only the farriers. There was a time, moreover, when surgeons were considered a bit like lackeys and bonesetters. We are far from these appreciations; today, in your ranks, famous names are mentioned. And now, that one of you* (Bouley) *becomes president of the first Academy in the world*.” If the profession owes a lot to Pasteur, the converse is also true which led Pasteur to comment: “*If I was young, and even at my age if I were able-bodied, I would go and become a student of the Alfort school. Reading veterinary books set my head on fire*” (51). Actually, the relationships between all six of these individuals, who were foremost in shaping our profession, were somewhat more complicated and not entirely harmonious, as detailed in Supplementary File 3.

Pasteur inspired Lister, the surgeon to Queen Victoria; Lister introduced antisepsis to surgery. Equally significant, Pasteur trained a generation of teacher-followers, who were to achieve their own marks of distinction, advancing knowledge on infectious diseases by developing vaccines, sera, and diagnostic tests. Nocard (1850–1903), professor of surgery at Alfort, identified several bacteria causing animal diseases, as well as the first mycoplasma (a pathogen in bovine peripneumonia). He further demonstrated that human and animal tuberculosis were the same disease, challenging Koch's view that tuberculosis of cattle was of no danger to humans (50). Also contrary to the view of Koch, it was shown that tuberculin could be used as a diagnostic tool (49) (Figure 5).

During the 1920s Gaston Ramon (1866–1963), a veterinarian from Alfort, was recruited by Emile Roux, the right hand-man of Pasteur. Ramon discovered anatoxins and established the role of adjuvants for vaccines. He made major contributions to the development of effective vaccines for both diphtheria and tetanus (52). Camille Guérin (1872–1961), also a veterinarian from Alfort and a pupil of Nocard, developed with Calmette the eponymous Bacillus Calmette-Guérin (BCG), a vaccine for immunization against tuberculosis (53).

In Germany, Robert Koch (1843–1910) identified the bacillary etiology of tuberculosis, the nineteenth century scourge of humans. His associate, Johann Wilhelm Schütz (1839–1920), professor of pathological anatomy and bacteriology at Berlin Veterinary College (54), was an associate of Koch, as Nocard was an associate of Pasteur (55).Together with Friedrich Loeffler he obtained, in 1882, a pure culture of *Burkholderia mallei*, the causative agent of glanders. With Robert Koch, Schütz also discovered the porcine glanders bacillus and demonstrated differences between the bovine and human tuberculosis pathogens. Koch was the first, in 1882, to introduce bacterial cultures. This was the first laboratory test to confirm bacterial infections. Koch also replaced gelatin with agar as a culture medium. In 1887, Julius Petri (1852–1921), a German bacteriologist and an assistant of Koch, replaced the glass plate with a circular, lidded culture dish; the Petri dish enabled isolation and observation of colonies whilst avoiding contamination. The Gram stain was introduced in 1884 by Hans Gram (1853–1938), a Danish pathologist and pharmacologist, while examining lung tissue from patients who had died of pneumonia (56).

In the UK, McFadyean, who graduated in 1876 and became the dean at the Royal Veterinary College in London in 1892, recognized immediately the significance of the germ theory of infectious disease and played a central role promoting research in the UK (57).

The Bureau of Animal Industry was established in the USA in 1884 under the direction of Daniel Elmer Salmon (1850–1914) (31). Salmon was the first US awardee of the degree of Doctor of Veterinary Medicine, from Cornell in 1876. Salmon also studied at the Alfort Veterinary School. Salmonella, was named after him in 1900 by Joseph Leon Lignières (1868–1933), a co-worker of Nocard at Alfort.

### The Pasteurian Revolution Created a New Basis for Therapy

The Pasteurian revolution was not limited to enhancing scientific knowledge and providing new therapeutic options, such as vaccines. Science also provided the basis for implementing strategic group animal therapies, based on epidemiological data. Solutions to public health issues were initiated through implementation of sanitation policies. This contrasted with and challenged traditional veterinary medicine using phytotherapy. Phytotherapy was directed primarily to individual animal medicine, especially in the horse. Individualized equine treatment, widely practiced in large cities and in the army, was shared between veterinarians, farriers and charlatans, with domination by locomotor conditions and a limited number of surgical procedures, such as castration. The continuing primacy of the individual horse failed to reflect the spectacular progress made in the control of infectious diseases.

Advances arising from the availability of vaccines emphasized the virtually total inadequacy of Materia Medica. This came close to acknowledgment in the 1910 edition of the Finlay Dun text, in noting, “*Cattle plague, contagious pleuropneumonia, rabies, and sheep-pox have been exterminated. The prevalence of glanders or farcy, swine-fever, anthrax, and bovine tuberculosis are also greatly limited by the measures now being adopted in dealing with these disorders*” (58). Materia Medica products could not lay claim to equivalence in terms of therapeutic success. This was despite progress in isolating and synthesizing the active principles of plants; and despite pharmacodynamic properties of the actives described in experimental models.

The absence or poor therapeutic results provided by Materia Medica relates to the near total lack of knowledge of pathophysiology. This prevented a rational use of substances, a minority of which did have therapeutic potential. For example, digitalis and its glycosides were used erroneously for treating fevers and inflammations and not for managing aspects of heart failure such as supraventricular arrhythmias, the pathophysiology of which was elucidated only later, when electrophysiological measurements became available. Likewise, the analgesic properties of morphine were well-known early in the nineteenth century but pain management in animals was not a clinical concern until the twentieth century and morphine (or rather opium) was used for digestive disorders.

Salicylic acid was synthesized in 1874; it was used as an antiseptic; in the late nineteenth century (59, 60) it was also used to treat rheumatic and joint diseases in horses but it was not significantly used as an anti-inflammatory agent for reasons explained in section: The Availability of Chemically Pure Drug Products Was Not Synonymous With Improved Therapy: see the case of Aspirin below. Ether was popular to treat bloat in cattle, being viewed as a narcotic purge. In 1840, ether and chloroform were used as vermicides for lungworm in calves (38). Ether, at anesthetic dose levels, was used to treat tetanus in horses; this use persisted during the nineteenth century.

The wide range of therapeutic uses of adrenaline, discovered in 1901, illustrates the lack of relevance to its actions. In a Materia Medica book published by Parke Davies and Co. in 1921 (61), 22 separate veterinary indications for adrenaline were listed with marketing in several formulations (solution, ointment, suppositories, and tablets). Most of these uses now seem bizarre. Examples are the treatment of rickets in puppies by intramuscular injection (34) and milk fever in cattle (61).

In short, a large majority of substances listed in Materia Medica books of the nineteenth and early twentieth centuries were not administered for actually useful indications, whilst horses continued to be bled and purged vigorously and operated on without anesthesia (38). There were exceptions. An example was the use of available local anesthetics, such as cocaine (1884). Cocaine was promoted for anesthesia of the eye. It was also used for epidural anesthesia in dogs in 1885 and spinal anesthesia in 1898 (62). The use of cocaine as a local anaesthesic, which found considerable use in horses in World War 1, is an example of a rational application. The Army Veterinary Corps, serving the British Army in the First World War, dealt with 2,562,549 horse admissions to hospitals between 1914 and 1918. Cocaine transformed the relief of pain, deriving from wounds and surgery. “*All surgery, where possible, was performed under anaesthetics. Chloroform and cocaine were in abundant supply. This was a great advance”* (63). The introduction of procaine in 1905 extended advances in regional and local anesthesia (37).

## The New Scientific Spirit Promoted the Rise of Professional Veterinary Journals Reflecting On and Challenging the Secular Dogmatism of Materia Medica

Up to the mid-nineteenth century, knowledge dissemination was not based on scholarly journals but on books, and most teachers had no research activity. Lectures by “the god professor” were almost the sole source of information for students and the eighteenth and early nineteenth century teaching of therapy resembled that of a religion, with Materia Medica as the Bible but with little variation in message.

**Figure 6:**
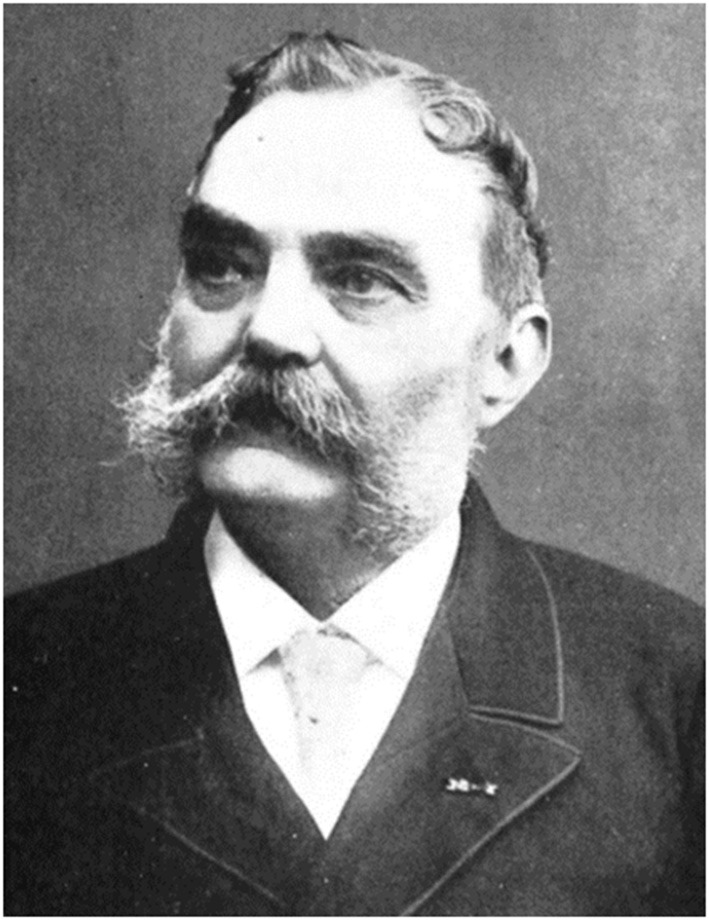
Alexandre Liautard, father of the American veterinary profession. Alexandre François Augustin Liautard (1835–1918), after obtaining his veterinary diploma from the National Veterinary School of Toulouse in 1856, emigrated to the United States in 1859. He moved to New York to practice the profession of veterinarian. At that time, there was no University in the USA to train veterinarians but Liautard founded a private college in New York (the New York American Veterinary College). Further, he assisted in the organization of the American veterinary profession and founded the United States Veterinary Medical Association, today the American Veterinary Medical Association. He was also the founder of the American Veterinary Review, now the Journal of the American Veterinary Medical Association (JAVMA). Liautard popularized in the USA the ideas of Pasteur, encouraging American veterinary medicine, then dominated by hippiatry, to adopt scientific approaches in the field of infectious diseases. As in Europe, this led to re-structuring based on science (see the Wikipedia article) (64).

However, the early to mid-nineteenth century brought predications of future change. The first “modern” professional periodical journals appeared. *Recueil de médecine véterinaire* based on the teachings of staff at Alfort (Paris) was launched in 1828. In the UK, following *The Veterinarian* (1828) and *The Veterinary Journal* (1844), *The Veterinary Record* (1888) became the primary information and reference source. In Germany, Carl Heinrich Hertwig and Ernst Friedrich Gurlt founded, in 1835, the ambitiously titled “*Magazin für die gesammte Thierheilkunde,”* (Magazine for the entirety of veterinary science). Publication continued until 1874. In the USA, a French veterinarian from the Toulouse veterinary school, Alexandre Liautard (1835–1918), emigrated to that country and founded the New York American Veterinary College (Figure 6). With others, he organized the American Veterinary profession, and co-founded the United States Veterinary Medical Association (USVMA), now the American Veterinary Medical Association (AVMA). He also founded, in 1863, the American Veterinary Review, now the Journal of the American Veterinary Medical Association (JAVMA). According to Freeman, Liautard, probably did more over a period of 40 years to advance veterinary medicine, practice, education and journalism than any other individual (65).

The contents of these journals differed from their present day successors. Specialists and researchers did not dominate them. Rather, they largely reflected practitioners' experiences, observations, and verbatim reports, including accounts of effects (or lack of effects) of remedies. Using current terminology, these journals comprised collections of case reports based on the practitioners' testimony.

Through these contexts, these Journals assisted the transition from Materia Medica and therapy to pharmacology and therapeutics as we currently recognize them. In 1889 at Turin, Lorenzo Brusasco, a Professor of Clinical Pharmacology at the Royal High School of Veterinary Medicine of Torino, published the “*Trattato teorico-pratico di materia medica e terapeutica veterinaria (farmacologia clinica): basato specialmente sui recenti progressi della scienza, ad uso degli studenti e dei veterinari pratici*” (Practical-theorical treatise on Medical Matters and veterinary therapeutics (clinical pharmacology) (66), especially based on recent science advances, to be used by students and practitioners). Like most veterinary pharmacologists at the end of the nineteenth century, he discusses the importance of the experimental approach in the field of physiology and therapeutics as the sole means for establishing a science-based discipline. He also recommended the use of pure active principles, in preference to extracts or raw materials. He discussed “kinetics,” laid special emphasis on administration routes and attempted to classify antagonisms based on mechanisms of drug action. His book included a chapter on posology, based on species size and age. Drugs were classified according to their action and/or use for the treatment of diseases : antiparasitics, disinfectants, antiseptics, analeptics, sedatives, and anesthetics. Maurice Kaufmann (1856–1923), at Alfort, authored the first French books on veterinary pharmacology and therapy, extending to four editions. The first edition (1892) was entitled “*Traité de thérapeutique et de matière médicale vétérinaire*” i.e., “*Treatise on therapeutics and veterinary medical matters*” (67). For the last edition (1910) the title was changed to “*Traité de thérapeutique vétérinaire: pharmacodynamie, pharmacothérapie*”, “*Veterinary therapy treatise: pharmacodynamics, pharmacotherapy*.” (68). In the USA, Kenelm Winslow published seven editions of his “Veterinary Materia Medica and Therapeutics” from 1901 to 1913 (69). In 1910, the 12th edition of the Finlay Dun text, “Veterinary medicines their actions and uses” was revised by J. MacQeen and H.A. Woodroff (58).

Unlike earlier texts, these books did not deploy the alphabetical dictionary approach. The presentation of remedies called for a degree of “therapeutic” logic and used citations of scientific works justifying efficacy claims. Kaufmann' book began with a chapter on general pharmacology, incorporating elements of what we now describe as pharmacokinetics. Absorption through the skin of a horse of iron cyanide and its excretion within few hours in urine was cited. In 1901, Winslow defined Pharmacology as embracing two sub-disciplines, Materia Medica and Therapeutics. Further, he distinguished between Rational Therapeutics, founded on physiological actions, and Empirical Therapeutics, based on clinical evidence. In the Introduction to the first, 1953 edition of his landmark book, Meyer Jones stated “*The various drugs are considered, first, with regard to their basic pharmacology (pharmacodynamics) and, secondly, to the clinical application of this information (therapeutics)”* (2). He thus firmly established that the evidential base for therapy should be the observation of and measurements on patients in the clinic and not the “no evidence therapy” peddled in earlier books.

## Evolution of Pharmacology as An Independent Discipline in Human Medicine and Its First Chemotherapeutic Success: The German Connection

**Figure 7:**
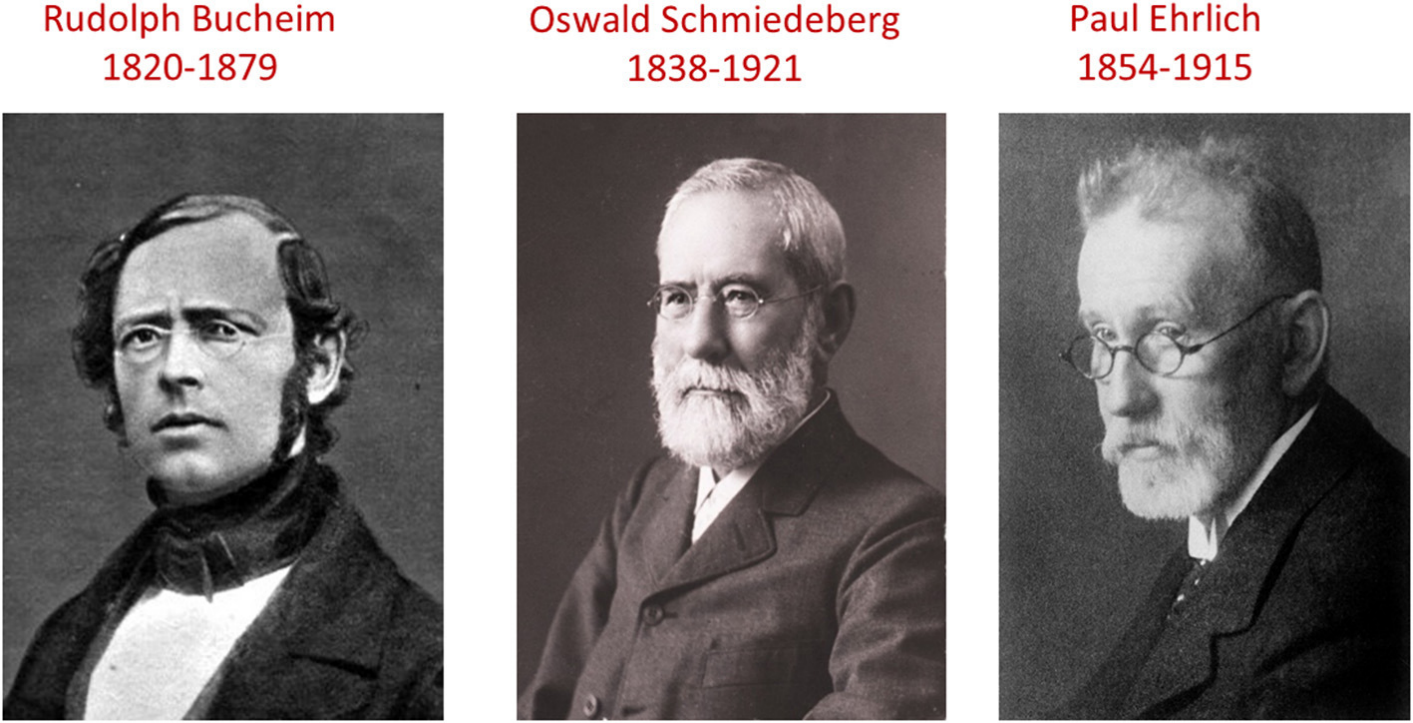
Buchheim, Schmiedeberg, and Ehrlich and the foundation of modern pharmacology. **(Left)** Rudolf Buchheim (1820–1879) was a German pharmacologist, now recognized as the “father of pharmacology.” He played a decisive role in the transition from Materia Medica to pharmacology by introducing quantitative *in vitro* tests (bioassays) and by his adaptation of the reference work of the time on Materia Medica by the Englishman Jonathan Pereira; Buchheim deleted the obsolete chapters. His pupil was Oswald Schmiedeberg (1838–1921) **(Middle)**. Of Baltic origin, Schmiedeberg was an emblematic professor of pharmacology at the University of Strasbourg. He is regarded as the “father of modern pharmacology.” His main contribution was to train many future world-renowned pharmacologists, including four future Nobel Prize winners. Paul Ehrlich (1854–1915) **(Right)** is the father of chemotherapy. He discovered arsphenamine (Salvarsan), the first effective drug treatment for syphilis. He also developed methods of staining pathogens, including the tubercle bacillus. Ehrlich's staining method was adapted to introduce the Gram stain, as it is still used today. In 1908 he received the Nobel Prize in Physiology or Medicine for his contributions to immunology, a prize he shared with Elie Metchnikoff (70).

The first teams devoted exclusively to the study of pharmacology in humans were those led by Rudolph Buchheim (1820–1879) (Figure 7) at Dorpat (now Tartu, in Estonia, at that time under Russian administration) and his student Johann Ernst Oswald Schmiedeberg (1838–1921) (Figure 7) at Strasbourg (Germany at that time). Buchheim was a founding father of human pharmacology. Of his two major contributions, the first was conceptual. He abandoned one of the major pillars of Materia Medica by dropping the old botanical and chemical classification of drugs and replacing it with one based on mode of action. In announcing the death of the “irritants”–numerous in Materia Medica–he said: “*We are used to deleting drugs from the series of irritants, as soon as we have gained some insight into their mode of action. Therefore, it is to be expected that, with increasing knowledge, the number of irritants will decrease until the term will fade eventually from pharmacology itself*.” Second, he introduced methods, such as the bioassay, for measuring quantitative responses, which were to become fundamental to advances in pharmacology. For a review on Buchheim see (71).

Buchheim's philosophy and working methods were promoted by his pupil, Oswald Schmiedeberg (1838–1921) and he brought world-wide recognition to the discipline (72). Schmiedeberg attracted some 120 students from 20 countries, creating “The Schmiedeberg School” of pharmacologists. His students, in due course, occupied forty pharmacology chairs, of which four became Nobel Laureates: George Hoyt Whipple (1934), Otto Loewi (1936), Corneille Heymans (1938), and Carl Ferdinand Cori (1947). Together with Bernhard Naunyn, Schmiedeberg founded the *Archiv für experimentelle Pathologie und Pharmakologie* (now *Naunyn-Schmiedeberg's Archives of Pharmacology)* the oldest journal of pharmacology still publishing.

Pharmacology cannot advance on the basis of concepts alone; it must also have active components, in the form of novel drugs. These, comprising chemotherapeutic agents, were supplied by Paul Ehrlich (1854–1915) (Figure 7). The common element underpinning his discoveries was the vital dyes and their *vivo* staining properties. In the last two decades of the nineteenth century, he showed that dyes react with specific components of blood and other tissue cells. He hypothesized that his *in vitro* observations might putatively lead to *in vivo* selectivity and, through this, to focused therapeutic uses. Specifically, he searched for the *magic bullets* i.e., chemical substances which have special affinities for living pathogenic organisms.

Ehrlich provided proof of concept with *Plasmodium falciparum* and *Plasmodium vivax*, the parasites responsible for malaria. They were stained with methylene blue, giving rise to its possible use to treat malaria. Results were promising in patients suffering from a mild form of the disease. This was the first report of a synthetic compound used successfully to treat a specific disease. There followed, in 1906, the first major discovery in pharmacology with clinical impact of the twentieth century: arsphenamine (Salvarsan or compound 606) was the first chemotherapeutic agent to treat syphilis.

However, this advance had no direct application in veterinary medicine. This came with the discovery of sulphonamides. Domagk's discovery of the antibacterial properties of the red dye, Prontosil, in 1935, was based on the survival of mice that had been inoculated with a highly virulent strain of haemolytic *Streptococcus pyogenes*. This was soon followed by recognition that Prontosil was a pro-drug, converted *in vivo* to sulphanilamide. Meyer Jones recorded this seismic shift in the first edition his textbook in 1953 (2) and we recently reviewed the origin and development of sulphonamides (5).

## The Availability of Chemically Pure Drug Products was Not Synonymous with Improved Therapy: The Case of Aspirin

Failure to identify correct doses for each species limited the early use of aspirin in animals. There were, however, additional limiting factors, including the advantage (if any) over salicylate and cost in a commercial setting, as considered by Winslow (34): “*It is merely a substitute (for salicylic acid) and is supposed to be less irritating to the stomach but this is not always the case… Fashion has at present endowed it with much wider scope than has been given to salicylates and salicylic acid. It is suitable in the case in which the latter are indicated, as in rheumatic affection and as intestinal antiseptic*.” Clearly, Winslow was not promoting the use of aspirin, which was an expensive drug, compared to salicylates. In screening veterinary handbooks for salicylate use, it is noted that salicylic acid was introduced to the Veterinary Pharmacopeia in 1880 and, in a Swedish handbook from 1892, salicylic acid was recommended for the treatment of fever and rheumatism. It was, nevertheless, considered to be “very expensive as 100 g are needed to a horse and 150 g to a cow per day” (73). The cost of treating a 600 kg horse with the correct dosage (100 mg/kg) would have been prohibitive. Today, aspirin is a very cheap drug but this was not so when launched by Bayer. Indeed, aspirin became the first “industrial drug.” It was, for commercial and societal reasons, reserved as a prescription medicine. At the end of the nineteenth century, advertising drugs directly to consumers was regarded as unethical and was strongly opposed by many medical organizations. Then, with the advent of World War One, phenol, essential for both the synthesis of aspirin and the manufacture of explosives, ran into short supply. By 1915, the price of phenol had risen to the point that Bayer's aspirin plant was forced to drastically reduce production. Aspirin then rebounded to enjoy great therapeutic success in humans during the Spanish influenza pandemic, of 1918–1920, as one of the most powerful and effective drugs available in human medicine. This was not the case in veterinary medicine, where aspirin was not only expensive but mainly regarded as an internal antiseptic rather than a pain killer because, at that time, pain was a diagnostic sign rather than a condition to be treated for animal welfare reasons.

## Chemotherapy in Veterinary Medicine Actually Commences with Antiparasitics

Prior to the twentieth century, treatment of animals' internal parasites was rudimentary; most Materia Medica medicines were either plant extracts or metals. It was assumed that they worked by mechanically irritating the parasites or by purgation of the host, to remove the parasite containing mucus from the bowels (74).The first efficacious antiparasitic drugs were of chemical origin. In the 1920s, Maurice Hall (1881–1938), in the USA, critically evaluated all resources then available, notably Materia Medica products, such as calomel, castor oil, magnesium sulfate, chloroform, ether, phenol, gasoline, male fern, areca nut, santonin, oil of chenopode, kamala, and many others. Most were judged to be either of no or very limited value (75). As chloroform was slightly efficacious, Hall tested carbon tetrachloride, which was also used as an anesthetic (76). Its efficacy in removing hookworms from dogs approached 100%. In the same year, Hall proposed the cautious use of this new remedy in human medicine for the treatment of hookworm disease (77). Although carbon tetrachloride is a potent hepatotoxin, several million people were treated. In 1925, Hall announced that tetrachlorethylene was just as effective but much safer, basing his conclusions on experiments in dogs. Tetrachlorethylene became the standard treatment for hookworm disease, both human and canine (75).

Phenothiazine, first synthesized in 1885, was initially used by Ehrlich for histochemical staining of plasmodia. The first therapeutic use, in 1934, was based on its lethal action on mosquito larvae. In 1938, the anthelmintic properties against intestinal parasites of pigs (2), and thereafter in other species, was shown. The introduction of phenothiazine as a veterinary anthelmintic provided the first compound with both broad spectrum and acceptable tolerance in host animal species (74). With phenothiazine came the important concept of control measures in veterinary parasitology, providing a rational strategy for prevention at the herd level, rather than individual curative treatment. This required detailed knowledge of sources of infection, means of transmission, life cycles, and parasite-host relationships (78). Phenothiazine had low potency, thus requiring very large doses. In due course, it was replaced by more potent anthelmintics such as thiabendazole (1961).

## Hormones, Receptors, and Neurotransmitters: The Basis of Functional Pharmacology and Therapeutics: The British Decades

Most hormones and neurotransmitters were discovered during the early decades of the twentieth century. However, these discoveries, unlike the vaccines, antiparasitic drugs, and sulphonamides impacting greatly on veterinary medicine during the first half of the twentieth century, did not find significant veterinary applications.

In the early 1900s, a fundamental question addressed was, how did communication across synapses occur, as electrical impulses or through chemical mediators. Otto Loewi (1873–1961) demonstrated in classical studies that acetylcholine, previously discovered in 1914 by Henry Dale (1875–1968), was an endogenous neurotransmitter. Next, came the understanding that, for a given neurotransmitter, there exist several receptors and, in consequence, several response outcomes. Moreover, it became clear that some plant extracts, or contained compounds, could block the transmitter actions. For example, Dale demonstrated both vasoconstrictor and vasodilator actions of adrenaline, the latter revealed by prior administration of an α-adrenoceptor blocker, ergotoxine, present in ergot of rye (79). For these discoveries, Levy and Dale were awarded the Nobel Prize in Physiology and Medicine in 1936. In the 1920s, Alfred Joseph Clark published his now classical papers on neurotransmitters, hormones, and drugs, demonstrating their interaction with receptors. Clark conducted important experiments on the action of acetylcholine on the heart muscle of the frog, and on the antagonism to this action by atropine. By recording the isometric contraction of the isolated strip of the frog's ventricle, he was able to show that the action produced was accurately expressed by the equation *Kx* = *Y*/(100 − *Y*) where x is the concentration of the drug and Y is the action produced expressed as a percentage of maximal possible action. Clark introduced the log concentration-effect curve, relating the hyperbolic shape of the stimulus-response curve to the equilibrium binding equation. He concluded that the concentration-action curve of acetylcholine reflected an adsorption process of the type described by Langmuir. Clark provided the first quantitative study of the antagonism of acetylcholine by atropine when he described the parallel shift to the right of the log concentration-response curves produced by competitive antagonists. He and his contemporaries also sought to estimate ligand uptake by organs using methods that represented the forerunner of radio ligand binding assays. Clark in 1933 published his Mode of action of drugs, the purpose of which was “to try to discover what laws could be postulated regarding the combinations formed between drugs and cells” (80).

The discovery of neurotransmitters [acetylcholine, norepinephrine (noradrenaline)] and of several classes of receptor for a given neurotransmitter enabled deciphering of the physiology of the autonomic nervous system, albeit drugs acting on them were not of major veterinary interest. However, the development of agonists and antagonists of increased selectivity and potency, and of adrenergic and cholinergic receptor sub-type classification found veterinary applications in the second half of the twentieth century.

In 1902, British physiologists, William Bayliss (1860–1924) and Ernest Starling (1866–1927), discovered secretin, thus introducing the concept of hormones, endogenous messengers secreted by one organ to affect remotely the functioning of another organ or system. Jacob Abel (1857–1938) of the Johns Hopkins University in Baltimore purified in 1899 the adrenal gland extract's active ingredient, epinephrine. Jokichi Takamine (1854–1922), a Japanese biochemist and successful business person, isolated the pure, stable, crystalline form of epinephrine, which he named adrenalin. The purified product was patented by Parke-Davis & Company in 1901. Accordingly, epinephrine was considered as the first isolated and synthetized hormone (81). For veterinary medicine, the only rational use of epinephrine was in combination with local anesthetics to prolong their action by delaying the absorption rate through constricting blood vessels at the site of injection (2).

The discovery of insulin in 1922 by Frederick Banting (1891–1941) in Canada, found immediate therapeutic application for the hormone extracted from bovine and pig pancreas. This transformed the therapy of diabetes mellitus. Banting was awarded the Nobel prize in 1923, together with Macleod (1876–1931). In veterinary medicine, several decades elapsed before formulations of insulin became available and prescribable for dogs and cats. This occurred when more advanced knowledge of diabetes mellitus became known in the 2000s. For example, in dogs there is no evidence for a canine equivalent of human type-2 diabetes (82) whereas type-2 diabetes is the most frequent form in cats (83).

In 1946, Edward Kendall (1886–1972), who had previously isolated thyroxine, now isolated four steroids from adrenal extracts, naming named them compounds A, B, E, and F. Compound E, cortisone, was synthesized later that year by Sarett (84). Its therapeutic potential was discovered by the rheumatologist Philip Hench (1886–1965) in a rheumatoid arthritis patient and then in many other inflammatory conditions. Hench and Kendall were awarded the Nobel prize for Medicine and Physiology in 1950.

In equine medicine, arthritic lameness has been treated with some success with cortisone, a pro-drug of hydrocortisone (cortisol, the natural hormone) injected intramuscularly or locally (intra-articular) (2). Cortisone was investigated in cattle for ketosis in 1951 (85) but the cost was prohibitive and the first veterinary products were synthetic analogs, discovered by the company, Schering (86).

Progesterone was isolated and characterized in 1929 (87) but its rational use, as well as those of other reproductive hormones, was delayed for several decades before integration into a global approach to control and synchronize reproductive cycles in ruminants in the 1960s (88).

## Effective Dosage Regimens: Challenges for New Veterinary Drugs

When veterinary medicine was on the brink of introducing single compounds of high purity, it had to resolve several generic problems that had not been addressed by Materia Medica. Primary considerations were setting dose regimens and dosage differences between species. In Materia Medica, species differences were generally attributed to idiosyncrasy (see later for morphine as an example) and not, as now, to differences in pharmacokinetics (89).

In France, Cerbelaud (90) proposed dosage regimens based solely on body weight, taking the unit of reference as dosage in man, which he equated to a large dog. For other species, dose was determined using a multiplicative factor (12 for cattle, 10 for horses, 5 for sheep, 2 for pigs, 1/4 for small dogs and cats, and 1/8 for chickens) (91). This approximates to an allometric relationship with a power of 0.75.

The science of pharmacokinetics provided an alternative and revolutionary basis for dose selection. In this, the introduction of sulphonamides in the 1930s was pivotal. Microbiologically based analytical techniques for measuring, with acceptable accuracy, the time course of active principle concentrations in blood, became available. However, it was not until the late 1970s, with the introduction of liquid chromatography, that veterinary pharmacokinetics advanced at a rapid pace, to be followed by clinical pharmacokinetics.

## Posology, Metrology, and the Apothecary System: Factors Limiting the Identification of Correct Drug Doses

Whilst the discovery of new active principles, and their synthesis on an industrial scale, were key factors driving the evolution of veterinary pharmacology, one factor which hindered its development, in English speaking countries, was the apothecary system of measures. This was based on the avoirdupois system. In metrology, as late as the 6th edition of Meyer Jones textbook in 1988 (92) the system of weights and measures applied to the preparation and administration of drugs was the apothecary system.

On the other hand, in dispensing prescriptions, the Troy grain and ounce were used as units of weight. The grain as a unit of mass was considered suitable for decoctions and similar formulations. However, it was not a convenient measure for expressing the dosage of potent drugs such as morphine. Rossoff (93) gave the dosage of morphine in grain fractions as 1/4 gr/10 lb. body weight. This corresponds to today's more readily comprehensible dose of 0.1 mg/kg body weight.

When ordering large quantities of drugs and in buying drugs without prescription, the avoirdupois pound of 16 ounces was used. The Troy pound is 5,760 grains (≈373.24 g), while the Avoirdupois pound is 21.53% heavier at 7,000 grains (≈453.59 g). The Avoirdupois ounce contains 437 grains, while the Troy ounce equals 480 grains. Nevertheless, the grain has same value in both systems. In the Troy system 1 pound=12 ounces = 96 drachms = 288 scruples = 5,760 grains.

To add further to these complexities, in the apothecary system, the unit's symbol or abbreviation is followed by the quantity in lower case Roman numerals. For amounts <1, the quantity is written as a fraction, or for one half (or semi-the Latin word for one-half or variations such as ss, ṡṡ, or $\bar{ss}$). Therefore, a prescription for tablets containing 325 mg of aspirin and 30 mg of codeine can be written “ASA gr. v c cod. gr. ss tablets” (using the medical abbreviations ASA for aspirin, c for “with”, and cod. for codeine).

The use of both Troy and Avoirdupois systems is utterly confusing for the modern mind. By contrast, the more logical metric system introduced by Napoléon and promoted by Moiroud in 1835 (45) uses Arabic numerals and decimals to indicate drug quantities. This metric system was adapted by Hertwig (39), but it was still set in relation to the old weights that showed some variability between the different federal states in Germany.

## Causes of the Demise of Materia Medica

### Failure Was Not a Problem in Itself, Either for Hippiatrists or Empiricists

By contemporary judgmental criteria, Materia Medica was, even in its ascendancy, a failure. According to Meyer Jones (2), the main issue was that “*Through his expert knowledge regarding the use of various plants and animal products in the treatment of disease, the medicine man became a very powerful individual in the tribe.”* Beliefs and legends, established over and unquestioned for centuries, justified to practitioners, through the nineteenth and into the twentieth century, continuance of the beliefs in the medicinal uses of plant-, mineral-, and animal-derived products. Each generation passed on the false folklore of their predecessors.

### Plants Are Complex Mixtures of Different Substances With Irreproducible Content

Most Materia Medica remedies were ill-defined. Plants are complex mixtures of substances with differing, even antagonistic, properties. For example, ergot of rye which was used as an oxytocic (44) or to induce abortion (67) contains many alkaloids. It is now known that they have affinity for several receptors. For the monoamine neurotransmitters, serotonin (5-hydroxytryptamine), dopamine, adrenaline (epinephrine), and noradrenaline (norepinephrine), there is a range of activities, which are often difficult to predict (94). Depending on type of plant, the raw material contents can be very variable with growing conditions on differing soils or in differing regions and harvest conditions. There was no consistency of either content or strength and, therefore, there could be no guarantees of efficacy, safety, and stability. For example, the active compound contents in Digitalis species are linked to seasonal variations of light intensity, the photoperiod, as well as the thermoperiod, soil nitrogen content etc. (95). In processing, these plants were subjected to the several potentially destructive processes of extraction, maceration, fermentation, heating, and conservation. It therefore was not possible to know both the strength of active and what adulterants were additionally being administered. Some authors of Materia Medica texts were aware of these factors. Hertwig (39) wrote in the chapter “modifications by the drug itself” that modifications are due “*to the location of the medicinal plant and its climate, the time and method of collection of the plants, the extraction and processing of their preparations, and their storage*” (39).

Theriac was regarded for two millennia, up to the mid-eighteenth century, as an universal panacea. It is an example of a “magic” concoction containing a multitude of plants and other ingredients, including viper flesh. Its preparation required essentially secretive methods, which extended over several months. Effect was wholly unpredictable but its preparation provided opportunities for competition between apothecaries, each having a recipe claimed to be better than that of colleagues. Reservations concerning the quality of Theriac and similar products prompted demands for their preparation in public, an early precursor of inspection of manufacturing process and premises and examination of the finished product. These developments led to the publication of pharmacopeias, in which standards for ingredients and compounding were laid down (96). The fact of having chemically defined substances was not, however, in itself, sufficient to secure their immediate adoption in veterinary medicine. Hering and Weiss (40) commented on Theriac: “*the so-called theriac, which used to be much abused in veterinary medicine, is a mixture of a lot of different plants … with opium, honey and the like. It deserves to be forgotten altogether*.” Theriac was similarly condemned by Hertwig (46) and not mentioned at all in the final edition of his book (39).

### To Be Used Rationally Many Remedies Would Have Required Pathophysiological Knowledge or Societal Values That Did Not Exist

Historically, plants were not, for the most part, used with any knowledge of their actions on physiological pathways. Lack of physiological/pharmacological knowledge meant that disease treatment was generally irrational and inappropriate. For example, decreased heart rate in response to digitalis glycosides was recognized by White (97) but he misconstrued the clinical benefit that this might confer; he predicted that they would be successful in treating fevers and inflammations of internal organs of the horse. This is in line with medical theories of John Brown (1753–1788), a Scottish physician, who claimed that all disease was caused by an unbalance of “*excitability*” which determined the body's ability to react to stimuli. The partisans of this doctrine regarded digitalis as a counter-stimulant par excellence. It, therefore, was used consistently in treating many inflammatory diseases.

For other compounds, such as morphine, societal values of the time failed to ensure its full potential for pain management in animals. Moreover, pain, in particular that of childbirth, was seen as a redemptive punishment, not deserving of pharmacological intervention. In the same vein, the use of anesthetics in humans was challenged, because surgery was to be reserved for virile men, with great manual skills and general anesthesia threatened these skills. However, there were dissenting, pioneering opinions; Hering (1870) (40) was an enthusiastic supporter of the pain relief provided by opium/morphine. He cites Andreas Christian Gerlach (Professor in Hannover): “*Surgical exercises should no longer be performed without this subcutaneous opium anesthesia, which is so easy to administer cheaply and safely*” *From now on, this will no longer be done at the local institution (Hanover)*.” Nevertheless, the recommended dose (1–1.25 g of morphine hydrochloride per horse) might be accompanied by side-effects, dysphoria and excitement. It was proposed to administer morphine hydrochloride “4–6 h before surgery” (see section Posology, Metrology, and the Apothecary System: Factors Limiting the Identification of Correct Drug Doses).

### The Notion of Experimental and Clinical Proof Did Not Exist and the Principle of Authority Reigned Supreme

The doctrinal views of the great ancients (Aristotle, Galen, and Dioscorides) were upheld with little dissent well into the nineteenth century. The clinical philosophy of what can be seen, prevailing over what should be investigated, was mitigated, but only rarely, in the nineteenth century. The consequence was delay in the introduction of therapeutics.

Why it took so long for common sense and visible evidence to prevail is difficult to comprehend, for example in the case of simple asepsis (Semmelweis 1818–1865). Difficult, likewise, to understand why blood-letting was long continued after the demonstration by Pierre-Charles Louis (1787–1872), not only of its ineffectiveness but of the associated dangers (10). Louis challenged blood-letting to treat inflammation, despite a perceived logical rationale, by introducing statistical reasoning in epidemiology. He promoted the so-called “*numerical method*.” In so doing, he was (what would be called today) a “pre-formal” epidemiologist. He based his “recherches” on the two key principles of epidemiology, namely group comparison and population approach (10).

Essential to progress in clinical pharmacology was the introduction of clinical trials and epidemiological studies. The first examples of “scientific” trials, of therapeutic measures known at that time, were those of James Lind on the effect of lemons in scurvy (1753), of William Withering on the foxglove (1785), and of Edward Jenner on vaccination against smallpox (1798). The Pouilly-le-Fort trial in 1881 to test the anthrax vaccine is an example of methodologically rigorous testing (see Supplementary File 3).

### The Notion of Correct Dosage Is Not Understood and Inter-species Differences Are Interpreted in Terms of Idiosyncrasy

Morphine is a classic example of why the rational use of a pure substance was not possible, in consequence of the failure to establish dosage regimens on a species basis. Morphine was isolated by Sertürner (1783–1841) in 1803–1805 (98) and commercial production by Heinrich Emanuel Merck, founder of the dynasty of the world's oldest drug company, commenced in 1827 (99). However, until relatively recently, veterinary medicine failed to match the actions of morphine with rational usage. Establishing both correct dosage and appropriate indications as an analgesic, advancing from paregoric elixirs known and used for their anti-diarrhoeal and antitussive properties, to the current sophisticated management of pain, were long journeys.

In 1913 Winslow (69) wrote: “*It is necessary to study the drug from the comparative standpoint in order to obtain a full understanding of its effect (), the brain of man, being more highly developed and sensitive than the brain of lower animals, it follows that this organ is more powerfully influenced in man and the action upon the horses and ruminants is something between that exerted upon the frog and the man.”*

Based on these considerations, the dose of morphine proposed by Winslow for the horse by subcutaneous administration ranged from 3 to 6 gr. (grains), ~0.3–0.6 mg/kg body weight, compared to a now recommended correct dose of 0.1–0.3 mg/kg body weight, which is the same as in man. He reported that the large dose of 12 gr. (1.2 mg/kg body weight) was followed by increased excitement aggravated by noise. Not surprisingly, Winslow concluded that “*the action of opium* (or morphine) *upon the horse differs from that upon man and dogs in the more frequent occurrence of restlessness and motor excitement.”* But he concluded that “*the rationale of the latter phenomenon has not been discovered.”* In other words, the choice of dose was made on (erroneous) *a priori* considerations of anatomy and phylogeny of the brain and not through empirical observation of its effects. His dogma overrode his observations.

Equating the cat, weighing of the order of 4 kg, with a small dog, gives a morphine dosage of 1/8–1/2 gr. (~1.875–7.5 mg/kg body weight). The dose currently recommended is 0.10–0.12 mg/kg body weight. A “*morphine mania*” syndrome, characterized by marked excitation, was reported and wrongly explained in terms of species-specific pharmacodynamics, termed species idiosyncrasy. This misconception was later reinforced by Wickler (100) who administered doses as high as 15 mg/kg body weight. In 1957, a Nature publication reinforced this falsehood; “*Since morphine produces a peculiar excitant or manic state in cats, we were interested in the possible influences of these tranquillizing drugs* (chlorpromazine) *on the morphine reaction in this species*” (101).This confirmed the earlier judgements–do not administer opioids to cats, at least not alone.

Even the 1965 edition of Meyer Jones book (102) continued the dogma, stating “*Opiates are clearly contraindicated in the cat, and they are rarely used in the horse*.” The 1965 edition contained no specific indication for treatment of pain. Rather, morphine was indicated for pre-medication prior to anesthesia, for the unwarranted aim of decreasing the amount of anesthetic administered. It was, moreover, recommended for the control of diarrhea and coughing. The inference is that the control of pain, and establishing an analgesic dose in animals, were not a basis for use from the animal welfare perspective. It is now recognized that “morphine mania” in the cat arose from much higher doses than are clinically appropriate. In the 4th edition of Meyer Jones in 1977 (103), it was proposed that the cat could be treated with morphine as an analgesic, with effective doses of 0.1–1.0 mg/kg body weight subcutaneously and 0.1 mg/kg body weight intravenously. In the 2018 edition edited by Riviere and Papich (104), the intravenous dose for cats is 0.1–0.25 mg/kg body weight with an inter-dose interval of 2–3 h.

That old concepts, however wrong, can be difficult to dislodge is illustrated by the introduction in the 1970s of a product containing the potent morphine analog, etorphine, in combination with the sedative, acepromazine. The dosage of etorphine was very high. After intravenous dosing, the horse was cast in a convulsant state, with front legs in spastic extension and hind legs in spastic flexion. The product also produced marked sympathoadrenal activation, with a two-fold increase in blood pressure and five-fold increase in heart rate (105).

### Therapeutics Education Suffers a Significant Time Lag Relative to Advancing Science

One motive in writing this review was a re-reading of the lecture notes in pharmacology taken by Calvert Appleby, an undergraduate student at the Royal Veterinary College, London, in the period 1946–1948. These provided a snapshot of the properties of drugs and how they were used in animals at a time of pivotal change. Albeit only reflecting instruction given at a single institution, they provide insights into the then still existing (in fact pre-eminence of) state of the art Materia Medica and therapy. Nevertheless, they were under challenge from the advancing science of pharmacology and its clinical application in therapeutics. Regarding the continuance of historical practices, the notes retain: the troy and avoirdupois systems of measurements; the naming of plants and drugs (including salts of metals) by Latin names; many classes of formulation and administration routes, from inhalation of anesthetics to rectal administration of drugs by suppository. Each drug is classified under headings of Origin, Characteristics, Administration, Specific Actions, Formulation, Dosage, and Uses. Descriptions are provided on drug-related legislation and prescription writing. The notes provide considerable detail on metals and their salts and propose many uses with, in almost every case, no evidential base.

On the other hand, the notes predicate the ascent of pharmacology and therapeutics, as illustrated by the introduction of sulphonamides and benzylpenicillin (penicillin G, used as both inorganic and organic salts), with descriptions of target pathogens, dosage and dosing intervals, adjusted for each species, according to plasma concentration-time profiles. There was recognition of the importance of attention to husbandry, diet, preventative measures, and the animal's general condition in treating disease. A range of factors, potentially modifying drug action, are outlined in the notes.

## Possibilities for the Resurgence of Materia Medica as for Herbal Medicine

In the twenty-first century, traditional medicine, deriving mainly from herbal medicine, retains a very significant component of modern medical care for most of the world's population. In Africa up to 80% of the population depend on local indigenous methods of healing, while 42% of those surveyed in the U.S.A. had sought alternative or traditional forms of health care at least once (106, 107). At least 25% of present-day medicines derive from plants and, recently, plant-based medicines have been found to be effective in the treatment of cancer (108), Human immunodeficiency virus infection and acquired immunodeficiency syndrome (HIV/AIDS) (109), and malaria (110).

The importance of traditional medicines in the developing world and their growing popularity in industrialized countries is not lost on the pharmaceutical industry and the medical research community. Both have sought to capitalize on the available knowledge.

The healthcare of animals has generally mirrored the use of herbal medicine in humans. For veterinary medicine, promoters of these new practices will be well-advised to be aware of metabolic adaptations in mammals, notably the major differences between herbivores and carnivores in respect of the metabolism of plant compounds. The emblematic example, with toxicological consequences for cats, is salicylic acid. Lloyd Davis described the species variability of salicylic acid pharmacokinetics, with its very short terminal half-life in herbivores (0.5 h) and the horse (1.0 h), longer in the pig (5.9 h), and dog (8.6 h) but much longer in cats (37.6 h) (111). These authors noted that the cat, unlike herbivores, does not have reason to ingest plants containing salicylic acid precursors.

### Why Do Plants Produce Compounds With Medicinal Uses but Which Are Poisonous to Animals?

Plants produce compounds with pharmacological and/or toxicological effects in humans and other animals. Is this coincidence, co-evolution or convergence? What are their roles in plant physiology? The most likely explanation is that plant-produced chemicals provide protection from predators, including animals, insects, and bacteria. The tobacco plant produces the insecticide, nicotine, and, emanating from this, have been the neonicotinoid group of insecticides (e.g., imidacloprid). Neonicotinoids are selective for receptors in insects compared to mammalian receptors. Whilst insecticides are less toxic in mammals, they are environmentally toxic, especially for the honeybee and wild pollinators (112).

Plants produce essential oils with antimicrobial properties (113). Recently, these have been rediscovered as alternatives to antibiotics (114). Plants also produce compounds e.g., alkaloids which are both bitter and toxic, providing the plant with a degree of immunity against attacks by herbivores and insects (107).

Thus, plants have evolved to produce compounds not involved in primary photosynthetic and metabolic activities, but which have protective properties against predators. They have been named secondary metabolites (115). The apparent lack of primary function, together with the observation that many secondary metabolites have specific negative effects on animals and lower organisms, including pathogens, has led to the protective hypothesis, based on repellence or toxicity (116). Metabolite production may increase when the plant is under attack, whilst some are released into the air in response to insect attacks. These compounds attract parasites and predators that kill herbivores. Metabolites providing defenses against herbivores are collectively known as anti-herbivory compounds. They include the many bitter alkaloids and tannins acting as deterrents to herbivores.

### Why Do Receptors for Secondary Metabolites of Plants Exist in Animals: The Example of Morphine

Morphine is a plant-derived metabolite, for which receptors exist in animals. Many plants produce compounds of the morphine class. The opium poppy genome has been defined in order to establish how it developed its therapeutic compounds (117). It is likely that morphine is a component of antimicrobial plant defenses, but the question remains, why do mammals have receptors for morphine. There are morphine receptors in the whale, a species that has no opportunity to ingest the poppy plant. In fact, the synthesis of authentic morphine is a general characteristic of eukaryotic cells, indicating a role for endogenous morphine (118) in animal cell functions. The widespread expression of morphine in plants, invertebrate and vertebrate cells/organ systems indicates a high level of evolutionary conservation of morphine and related morphinan alkaloids, as essential chemicals for normal growth and development.

Similar evolutionary conservation has been reported for ouabain. In 1991, Hamlyn et al. (119) isolated from human plasma a cardiotonic steroid, which was indistinguishable from plant-derived ouabain. It was named endogenous ouabain. Endogenous ouabain has been isolated from the bovine adrenal gland and hypothalamus and identified, by mass spectrometry and nuclear magnetic resonance, as identical with plant-derived ouabain. Postulated physiological roles include regulation of vascular tone and sodium homeostasis. The physiological role in plants is unknown.

### Paleopharmacology and Bioprospecting

Bioprospecting, also described as biodiversity prospecting, is the systematic and organized search from bioresources including plants, microorganisms and animals. The aim is to develop products for pharmaceutical, agricultural, cosmetic, and other applications. This concept is not new (3, 120) but it has gained recent popularity amongst academic and industrial researchers (121).

To avoid biopiracy and to overcome the risks and ethical controversies associated with bioprospecting in developing countries, one approach has been to revisit ancient European texts and extract from them information that the doctors and apothecaries of the time could not develop fully, but whose testimonies might be explained in the light of current knowledge. For example, researched remedies, described in the old herbal literature, may have potential for use in rheumatic conditions and chronic inflammatory and degenerative diseases (122). They identified 63 plants for these indications and more than half of them have been shown to possess, *in vitro* or *in vivo*, antiphlogistic activities.

Natural herbal remedies have been recommended, with claimed evidence of efficacy, which is almost universally lacking. For the future, herbalists must ineluctably face the question of variability in active compound content in plants, depending on source and batch differences, as well as adulteration with many other compounds, with no or even undesirable actions. Herbalists will also face the issue of justification for use of plant mixtures, contrasting with the assurance of selectivity provided by the well-characterized active constituents as pure drugs. To cite one example, anemia must be addressed by determining specifically the cause, such as immune-mediated destruction, blood loss, or bone marrow suppression and not just from a simple symptomatic approach. Possibly, blood tonics might offer temporary relief, whilst the cause is addressed, and may even stimulate hematopoiesis. An approach might consist of administering a combination of blood tonics (Rehmannia and Dang gui). For non-specific conditions, adaptogens (such as Rhodiola or Ashwagandha) have been promoted to increase resistance to several types of stress and fatigue and to improve immune health. However, the European Medicines Agency reported that “*the principle of an adaptogenic action needs further clarification and studies*” and therefore the term “adaptogen” cannot be used for marketing in the EU.

## Conclusions

Prosecuting the past in the court of the present is a task that should not be undertaken lightly. There is an historical case for the defense of Materia Medica, in that, for more than two millennia, there was virtual universal acceptance of the teachings of Galen, Dioscorides and others, handed down from generation to generation and century to century. The defense is bolstered, not so much by the limitations of the science of the day, but rather by the virtual absence of any scientific base at all. It was a case of virtually no science–and therefore commonly nonsense. In place of science were firmly held and rarely challenged religious beliefs and stemming from them a notion that either providence or God in his goodness would provide in nature materials for the treatment of diseases. The case for the prosecution of Materia Medica is that those profiting from it had no sense of the curious. Rather, they organized remedies on an alphabetical basis and not on any observed beneficial or any measured effect. This strict alphabetical order was questioned in the nineteenth and was abandoned in the twentieth century. Insofar as observed effect dominated, it was not one of efficacy but of profound visually observed effects in the treated animal. Bleeding, purging and other non-efficacious depletive and derivative methods were deemed to be the basis of cure. When the wide sweep of the scientific revolutions, notably the pharmacological and the Pasteurian, came into play, they superseded slowly but inevitably the dogma of centuries. The evolving sciences were initially retarded by practitioners' (whether qualified or not) reluctance to relinquish the basis of their livelihoods. The scientific revolutions could not be applied optimally until the instrumentation revolution enabled diagnosis of both causes and signs of disease. It was as late as mid-twentieth century that Materia Medica practices were finally replaced by the modern era of pharmacology and therapeutics.

## Author Contributions

PL and P-LT jointly conceived the idea, performed literature searches, extracted relevant data, and drafted and edited the manuscript through nine drafts. WB provided essential information on a German perspective and assisted with review of the manuscript through the last five drafts. All authors contributed to the article and approved the submitted version.

## Conflict of Interest

The authors declare that the research was conducted in the absence of any commercial or financial relationships that could be construed as a potential conflict of interest.

## Publisher's Note

All claims expressed in this article are solely those of the authors and do not necessarily represent those of their affiliated organizations, or those of the publisher, the editors and the reviewers. Any product that may be evaluated in this article, or claim that may be made by its manufacturer, is not guaranteed or endorsed by the publisher.
