# Supplementary material for: The Decline and Fall of Materia Medica and the Rise of Pharmacology and Therapeutics in Veterinary Medicine
Source: Front Vet Sci. 2022 Jan 20;8:777809. doi: 10.3389/fvets.2021.777809 (PMC8810541; doi:10.3389/fvets.2021.777809)
Supplement: Supplementary file 1 [file Data_Sheet_1.pdf]

Supplementary Material

Supplementary file 1

Veterinarians and farriers

For much of the 19th century farriers, quacks and qualified veterinarians were in competition and opposition. However, this did not apply in the British Army. In 1811-12, it was recommended that a Farrier Major, with the rank of Sergeant, be appointed to each regiment, '*his duty being the superintendence of all the Farriers under the direction of the Veterinary Surgeon*'. Veterinary Surgeon J. W. Gloag of the 10th Hussars described the role of the farrier in 1839. '*Every farrier when mounted was to be in possession of a phleme, blood stick, two linen bandages, some tow, and a clyster apparatus carried in a holster. Each troop had a chest of prepared veterinary medicines, with the use of which every farrier was expected to be well acquainted*'. Gloag was '*most impressive in urging the young practitioner not to bleed unless absolutely necessary, this at a time when the belief in bleeding as a necessary part of all treatment was universally held…[On glanders]…his disinfectant was chlorinated lime*' (1). In 1854 the (British) Army Veterinary Corps drug provisions for the Crimean War, comprising Headquarter Chests, were described by Smith (1). '*In the scale of drugs, among other items, 10 lbs. Resin, 12 lbs. Aloes and 7 lbs. Potassium Nit. were allowed for 50 horses for six months. Diuretics and purgatives were prominent*'.

1. Smith F. A History of the Royal Army Veterinary Corps 1796-1919. Bailliere Tindall and Cox; London 1927. 268 p.
