# Supplementary material for: The Decline and Fall of Materia Medica and the Rise of Pharmacology and Therapeutics in Veterinary Medicine
Source: Front Vet Sci. 2022 Jan 20;8:777809. doi: 10.3389/fvets.2021.777809 (PMC8810541; doi:10.3389/fvets.2021.777809)
Supplement: Supplementary file 2 [file Data_Sheet_2.pdf]

Supplementary Material

Supplementary file 2

Veterinary science and institutional conquests

In England, the Royal College of Veterinary surgeons continued to require no formal qualification until 1864. During the 1870s and the 1880s, George Fleming, a British army veterinarian, called on colleagues to involve themselves more with contagious animal diseases and the inspection of meat and milk. To be able to do so, he urged, veterinarians needed to become ‘educated scientific men’. However, the exclusive right to conduct clinical work and administer medicines according to therapeutic principles is of relatively recent origin. In France, the diploma of veterinary doctor after defending a thesis, as we know it today, was established by the Law in 1923 and it was only in the 1970s that the last empiricists disappeared, the exercise of which was made illegal in 1938. In the UK, it was not until 1881 that the Veterinary Surgeons Act restricted the term ‘veterinary surgeon’ to practitioners who had received formal training and obtained qualifications from a veterinary school. The mainstream profession was eager to differentiate veterinarians from unqualified ‘quacks’ by reforming the equine-focused veterinary curriculum to introduce ‘scientific’ subjects relevant to agriculture and public health (1). The Act notwithstanding, it remained legally possible to carry on a veterinary business, provided the individual did not claim the title of veterinary surgeon. In the United Kingdom the 1881 Act recognized the qualified veterinarian as the person legally entitled to conduct the practice of veterinary medicine, but it was not until 1948 that quacks, acting perfectly legally until that time, were debarred in the UK and into the 1970s in France. Moreover, in societal terms, during much of the 19th century, the veterinary profession was not consolidated. Even with their formal qualifications, registered veterinarians did not have much greater knowledge on which to base disease treatment than the quacks and farriers, with whom, in civil life, they competed. In 1828 Percivall described the condition of veterinary medicine in UK at that time. For details on the development of Veterinary Medicine in England see (2)

1. Skipper A. The ‘Dog Doctors’ of Edwardian London: Elite Canine Veterinary Care in the Early Twentieth Century. Social History of Medicine. 2019 Jun 14;hkz049.
2. Woods A. From One Medicine to Two: The Evolving Relationship between Human and Veterinary Medicine in England, 1791–1835. Bulletin of the History of Medicine. 2017;91(3):494–523.
