# Supplementary material for: The Decline and Fall of Materia Medica and the Rise of Pharmacology and Therapeutics in Veterinary Medicine
Source: Front Vet Sci. 2022 Jan 20;8:777809. doi: 10.3389/fvets.2021.777809 (PMC8810541; doi:10.3389/fvets.2021.777809)
Supplement: Supplementary file 3 [file Data_Sheet_3.pdf]

Supplementary Material

Supplementary file 3

The three early giants of microbiology (Pasteur, Koch and Lister) and "their associated veterinarians" (Nocard, Schütz & Toussaint)

Pasteur, Koch and Lister are the three outstanding figures of the 19th century concerning the origins of bacteriology and medical microbiology. Veterinary medicine was to immediately follow in the footsteps of these giants. Over approximately fifty years, from 1865 to 1920, it played a full part in the major discoveries which revolutionized both human and veterinary medicine. This gave to veterinary medicine its stature, a stature which empirical hippiatry could not provide. Many veterinarians participated in this scientific saga. Three, Nocard, Toussaint and Schütz, are highlighted here in recognition of having played leading roles, arising from their proximity to Pasteur, Lister and Koch, respectively. Toussaint played a central role in the early controversies involving Pasteur, with Koch in an intermediary role adding fuel to the fire.

Pasteur (1822-1895), a chemist by training, is the acknowledged founder of microbiology. His discoveries on spontaneous generation (1861-1862) fermentation (1863) and the microbial aetiology of diseases of silk worms (1965) were fundamental to the emergence of the germ theory of diseases. It was only at the age of fifty four that Pasteur became involved in the study of animal (anthrax) and human diseases (rabies) (1). However, his discoveries on fermentation had already influenced Joseph Lister (1827-1912) (2). Lister was the first person to apply the Pasteurian germ theory to human medicine (3), notably to surgery. Lister graduated from London as Bachelor of Medicine in 1852 and was appointed Professor of Surgery at the University of Glasgow in 1860. At that time, sepsis was the major factor limiting the development of surgery. Mortality rate, after amputation, was of the order of 50% at the London hospital between 1852 and1857 (4) and surgery was viewed as a lottery, as to whether patients

recovered or died. It was assumed that exposure to "bad air", or miasma was responsible for infections in wounds and, in the absence of any theory of bacterial infection, a surgeon was not required to wash his hands or sterilize materials before carrying out surgery (2).

Lister was intrigued by the fact open fractures almost always ended in gangrene, whereas closed fractures had a much better prognosis. He became aware of the "philosophic" work of Pasteur (5) on fermentation and putrefaction, immediately making the connection between suppuration and putrefaction. This led him to formulate the hypothesis that suppuration is equivalent to in vivo fermentation caused by the growth of micro-organisms and did not occur independently of their presence. In addition, this suppuration could be controlled, as suggested by Pasteur for putrefaction, with antiseptics. Lister wrote: '*In the course of the year 1864, I was much struck with an account of the remarkable effects produced by carbolic acid upon the sewage of the town of Carlisle, the admixture of a very small proportion not only preventing all odour from the lands irrigated with the refuse material, but, as it was stated, destroying the entozoan which usually infest cattle fed upon such pastures"* (6).

In March 1865, Lister operated on a young boy with a compound fracture of the femur, a generally disastrous circumstance in terms of prognosis. He used a 5% solution of carbolic acid (phenol) for "*the destruction of any septic germs which may have been introduced into the wound, either at the moment of the accident or during the time which has since elapsed*. *This is done by introducing the acid of full strength into all accessible recesses of the wound by means of a piece of rag held in dressing-forceps and dipped in the liquid*" (7). This first surgery with an antiseptic was successful. In 1867 Lister published his classical paper entitled "*On the Antiseptic Principle of the Practice of Surgery* " now reprinted as a classic article (8). The mortality rate in surgery was reduced to less than 10% after introduction of aseptic principles. They rapidly became widely adopted and Lister was accorded the status of father of modern surgery. More recently, some authors have questioned the impact of Lister's work on the reduction of mortality in surgery (9).

In 1878, Lister was appointed to the prestigious position of "Surgeon in Ordinary" to Queen Victoria. It was his status of Surgeon of the Queen which enabled him to work

at the National Veterinary School of Toulouse, where he conducted, with his friend Henry Toussaint, professor of physiology, experiments which were prohibited in Great Britain (vide infra) at that time.

In 1866, Robert Koch (1843-1910) had recently graduated in medicine in Prussia. The Franco-Prussian War commenced in 1870 and the defeat of France in that year was a terrible blow for Pasteur, who was very close to the imperial family (Napoléon III visited and supported his laboratory) (11). However, Koch was also a patriot and, in 1871, he enlisted in the German army as a volunteer surgeon to support the war effort against France. In 1872 Koch was discharged from the army and was then appointed as a district physician in Wollstein (Prussia, now Wolsztyn in Poland). Here, connecting with his patient room, he established a private laboratory, where he conducted research on microorganisms. Using only rudimentary equipment (his microscope was a gift from his wife), he developed techniques for isolating and growing selected pathogens in pure laboratory cultures. As a rural physician, Koch had the opportunity to study anthrax during an epidemic outbreak in sheep in his area of countryside. Casimir Davaine in France (1812-1882) had already discovered, in 1850, a microorganism (now known to be *Bacillus anthracis*) in the blood of diseased and dying sheep. Koch, like Davaine, believed that the anthrax bacillus caused the disease. Tracking the complete life cycle of anthrax bacilli in and out of the host, Koch discovered that, under adverse conditions, they form heat-resistant spores which lie dormant for long periods, only to germinate, multiply, and wreak havoc under favourable conditions. The stability of the spores accounted for the persistence of anthrax in pastures long thought to be cleared of the organism, thereby leading to unexpected flare ups.

In 1876, Koch published his discovery of the anthrax bacillus, after demonstrating that mice, inoculated with pure cultures derived from sick animals, died of anthrax. Thus, a specific pathogen could give rise to a specific disease, establishing for the first time relevance of the germ theory of Pasteur in the aetiology of an infectious disease (12) i.e. he was able to demonstrate what is now known as one of the Koch’s postulates, namely the causal relationship between a specific microorganism and a disease (13). Photography was a hobby for Koch and his publication in 1877 on the structure of the anthrax bacillus marked the first photograph of a bacterium (14). At the time, pure

microbial cultures were extremely difficult to obtain and some critics argued that Koch's result was inconclusive, because he failed to show that his culture of the anthrax bacilli was indeed pure. The dispute drew Pasteur into anthrax research who, at the age of fifty four, now orientated his research to diseases of large animals and humans (1). Pasteur filtered the bacillus culture, passed it through healthy experimental animals, and showed that the purified culture caused anthrax. Pasteur, in an 1877 publication, referred to Koch but argued that only his own method was capable of ensuring purity of the bacillus culture. Therefore, Pasteur claimed to be the first to demonstrate the causal agency of the anthrax bacillus.

Koch, as a rurally-based Doctor, was in no position to challenge Pasteur, who had already achieved iconic status. Nevertheless, the battle for anthrax had indeed begun. It was to involve a third protagonist, the veterinarian, Henry Toussaint (1847-1897). In December 1876, Toussaint, a pupil of the great physiologist Auguste Chauveau (1827-1917), who had graduated as a veterinarian from Lyon in 1869, was appointed as professor of anatomy and physiology at the Toulouse Veterinary School. Toussaint commenced his own work on anthrax in 1877. He presented two verbal communications to the Académie des Sciences de Paris. The Academy was, at that time, the hub of scientific exchanges in France and its 'Comptes Rendus' were read worldwide. Through this channel, Pasteur became aware of and closely followed the studies of Toussaint. Over several years, Pasteur and Toussaint exchanged many letters, Toussaint explaining his investigations to the iconic master and sending him biological samples for testing. Despite this apparently close cooperation, Pasteur, and especially his collaborators, remained very sceptical of the various discoveries of Toussaint; as they were unable to reproduce his results, they refuted his interpretations. In order to ensure his pre-eminence, Pasteur applied the precautionary principle, adopting for Toussaint the same suspicious behaviour with which he viewed Koch. He continued to regard himself as the founding father of the discipline, and was not prepared to contemplate sharing that role with others.

On July 12, 1880, Toussaint filed a "sealed envelope" with the Academy of Sciences. In this, he referred to the possibility of acquiring immunity against anthrax through an attenuated vaccine against anthrax in dogs and sheep (15). Because of the crucial importance of the communication, the secrecy was quickly lifted and the procedure,

which involved heating defibrinated blood at 55 °C for 10 minutes, was revealed. Toussaint further explained his use of heat or phenic acid to obtain his vaccine. Pasteur totally denigrated the Toussaint discovery. In August 1880, Toussaint attended the Cambridge Congress. During this event, Lister, after saluting "*the eminent pathologist Professor Toussaint of Toulouse, whom I am proud to see present in this Section to-day*", reported to the assembly the work of all three pioneers, Pasteur, Koch and Toussaint, on anthrax (16). Toussaint and Lister established a firm friendship. Lister used this to circumvent Queen Victoria's injunction on animal experimentation. Indeed, Queen Victoria, who was shocked by animal experimentation, forbade her personal surgeon to practice vivisection. Queen Victoria's repugnance for the conduct and teaching of animal experiments to students had previously been expressed in a letter to Lister, when he was Professor of Surgery in Scotland; her private secretary wrote ''*The Queen has been dreadfully shocked at the details of some of these practices, and is most anxious to put a stop to them*.''(17). The Cruelty to Animals Act was enacted in 1876 to regulate the conduct and conditions of animal experimentation. It is in this context that, in 1881, Lister went to the Veterinary School of Toulouse to conduct experiments on the pathophysiology of blood clotting, by placing small tubes in the jugular vein of a donkey; this was in relation to the infection process that he was studying. He reported his results in the Lancet (19 November 1881) indicating in the introduction that '*The experiment was of a character such as it would have been difficult under existing circumstances to perform in London, so I resorted to the Ecole Veterinaire of Toulouse, where every thing was most liberally placed at my disposal by my friend Professor Toussaint and others in authority at the institution'*. (18). Establishing that a blood-clot is an even less favourable nidus than serum for bacterial growth, he observed a multitude of similar corpuscles in microscopical sections of the walls of the veins. These corpuscles were phagocytes. Their phagocytic action, not yet known, was established in 1883 by Elie Metchnikoff (1845-1916) (19), a French biologist of Ukrainian origin who shared with Paul Erlich (1854-1915) the Nobel prize in 1908 for their respective contributions to cellular and humoral immunity. This double attribution recognised the duality of immune mechanisms and put to rest what German scientists, still suspiciously described as "the phagocytosis romance", which derived from France (19).

Meanwhile, Koch, in 1880, obtained a full-time research position in Berlin at the

Imperial Health Office, where he worked from 1880 to 1885. Having now adequate resources, Koch developed novel staining and culture techniques using agar and glass plates (later developed as the Petri dish by his assistant Julius Richard Petri) (20). In 1881, Koch published a reference book entitled "*Zur Untersuchung von Pathogenen Organismen*" (Methods for the Study of Pathogenic Organisms). This became known as the "Bible of Bacteriology" (21). The main methodological contribution of Koch was the replacement of fluid by solid media for isolating pure cultures of micro-organisms. Solid culture methodology was simple, clean and reliable; it revolutionized the study of pathogens and the methods are still used today (22). In 1881 Koch demonstrated his new technique at the International Medical Congress in London, and Pasteur "complimented him on his work" (20).

French researchers, including Pasteur, wished to avoid use of the term bacteriology, preferring the term microbiology to emphasize that the priority was to seek medical solutions to disease, rather than an understanding and refinement of the precise pathophysiology of the disease being treated. In this context, 1881 was the decisive year for Pasteur with his iconic demonstration of the efficacy of a vaccine in the prevention of anthrax, for which he publicly claimed (and was acclaimed for) his success. Anthrax devastated the countryside and this placed him under political pressure to find a solution. Pasteur took up the challenge to make a public demonstration of the effectiveness of his 'so-called vaccine'. The demonstration was organized by the veterinarian Rossignol, in Pouilly-le fort, near Paris. Rossignol himself, like most veterinarians, was unconvinced and, one month before the experiment, he had satirized the germ theory and Pasteur in the following terms "

Microbiolatry is the fashion, it reigns undisputed; it is a doctrine which must not even be discussed, especially when its Pontiff, the learned M. Pasteur, has pronounced the sacramental words, I have spoken (11). The demonstration was conducted using a robust protocol, previously agreed between Pasteur and the organisers. The protocol was a controlled clinical trial in 50 sheep, half to be vaccinated, half serving as controls, with all sheep receiving a lethal inoculation of anthrax, then examined after the time needed for development of vaccine protection. Pasteur accepted last minute modifications to the protocol (introduction into the trial of goats and a few cows). The experimental observation day was June 2, two days after the anthrax challenge. Invites to observe the animals were extended internationally and included the London

Times (3). Nocard (1850-1903) Pasteur’s veterinary colleague attended.
The climax was recorded in an Oscar-wining film (1). Pasteur was not at all sure of the result and he therefore took a considerable risk. To avoid ridicule, he waited wisely in Paris to see how events turned out. Pasteur was ready, in case of failure, to blame his closest collaborator, Emile Roux, who had prepared the vaccine (3). On the morning of June 2, Pasteur received a telegram from Rossignol informing him of a "stunning success". Pasteur rushes to Pouilly-le Fort and there, the legend was born. Pasteur’s zealots claimed that the ”Maître” (master) calmly approached the resting animals and that all the vaccinated animals stood up to greet Pasteur, leaving at their feet carcasses of the 24 unvaccinated sheep (1). The outcome was clear, with no requirement for statistical analysis. This was the legend, but the reality was less clear. It is now known that Pasteur gave a misleading account of the preparation of the anthrax vaccine used in the Pouilly-le Fort experiment. Lacking confidence both in his own “live” vaccine and his attenuated vaccine plus oxygen, he required his co-worker, Charles Chamberland, to quickly prepare another attenuated vaccine (1). In a summary report of his experiment, Pasteur did not provide a description of the methods of preparation of his vaccine and kept this confidential (23). But now we know that Pasteur did not use his own vaccine but one following the Toussaint formulation, in which carbolic acid was replaced by potassium dichromate (23). This subterfuge (today this would be a clear case of misconduct) was not made public until much later, 1938, when Pasteur's laboratory notebooks were made public by Adrien Loir, a nephew of Pasteur, who was his secretary (24). Toussaint was never able to gain the appropriate recognition of his critical contribution, because he was wholly out of action by 1883, at the age of 36, suffering from a severe cerebral condition from which he died in 1895.
It fell to Koch to reveal (in part) the truth. Koch, who had accounts to settle with Pasteur, did not wait to learn of Pasteur 's plagiarism (also known as « Secret de Pouilly-le-Fort »). He recognized Toussaint's contribution in an 1883 article entitled “*Ueber die Milzbrandimpfung. Eine Entgegnung auf den von Pasteur in Genf gehaltenen Vortrag*" (About the anthrax vaccination. Reply to the conference given by Pasteur 4th international hygienic congress in Geneva) and also in the Lancet (25). Koch wrote a long and vitriolic diatribe, in which he methodically attacked Pasteur's lack of rigour (26). He further boldly asserted that Toussaint, and not Pasteur, was the real discoverer of the anthrax vaccine. Moreover, Koch pointed to the fact that Pasteur was not a doctor, which might explain some of his shortcomings (21). He ended his evaluation saying: “*When Pasteur was celebrated as a second Jenner at the Congress of Geneva, it was probably somewhat premature, and in the rush of enthusiasm, it was apparently forgotten that Jenner's*

beneficial discovery was not for the benefit of sheep, but of people”. We are here far removed from the atmosphere of the Seventh International Medical Congress, held in 1881 in London, where Lister had managed to bring the French and German protagonists together, in itself a triumph, as Pasteur could never forget the war of 1870-71. As Pasteur took Koch's hand, Lister exclaimed: " C'est un grand progress” (27).

Following his discovery of the slow growing tuberculosis bacterium (Mycobacterium tuberculosis) in 1882, a discovery for which he was awarded the Nobel prize in 1905, Koch was promoted to Geheimer Regierungsrath, a senior executive position. At the time, tuberculosis was a scourge and the leading cause of human infectious deaths in Europe killing approximately 25% of the adult population (28). It was wrongly considered to be an hereditary disease, because it was often reported within families. Two French doctors Gaspard Bayle and René Laënnec (the promoter of pulmonary auscultation) discovered in 1803 that those who died of "consumption" presented at autopsy tubercles in all organs. This observation led in 1839 to a new name for this scourge, tuberculosis (1). Its contagious nature was demonstrated in 1865 by Jean-Antoine Villemin. He showed that the sputum of tuberculosis patients, inoculated into rabbits, induced a disease similar to tuberculosis (1). These findings were confirmed in 1882 by Koch, who further isolated and identified the causal agent, the tubercle bacillus (26).

Koch followed Pasteur into iconic status, with the expectation of discovering a therapeutic solution to the disease, which was the leading cause of death in adult humans at this time, with a long list of celebrity victims (1). However, the discovery of the tubercle bacillus, in contrast with many other infectious diseases, did not lead to a vaccine for many years. It was in the 1920s that the first acceptable vaccine was introduced in France by Calmette (a physician of the Pasteur institute) and Guerin (a veterinarian and pupil of Nocard). They gave it their name to “Bacillus Calmette & Guerin”, the now eponymous BCG vaccine (29).

It was against a background of expectation, impatience and pressure from government officials that Koch made a mistake that he came to regret (26). In August 1890, Koch mentioned at a medical congress that he had preliminary data on a substance, (the tuberculin) that halted the growth of tubercle bacilli in laboratory cultures and guinea pigs. One month later, he reported in a communication “*on a cure for tuberculosis*”. And when a world-class authority of Koch’s standing announced the tuberculin “cure”, thousands of patients migrated to Berlin (26). After further investigations, it was reported that a low dosage of tuberculin induced reactions in tuberculosis patients but

not in healthy people, thus predicting that tuberculin would be a simple and effective diagnostic tool for tuberculosis. Whilst this is correct, when tuberculin was administered at high dose rates as a remedy, very severe allergic reactions and deaths occurred frequently and tuberculin, as a cure, was discredited and the reputation of Koch tarnished (26).

In 1868, Edmond Nocard entered the Veterinary School of Alfort, where he was to spend most of his career. His studies were interrupted by his enlistment in the army in 1870, during the war against Prussia. In 1873, he became a veterinarian and then head of clinical service in Alfort. After having met Émile Roux, the physician collaborating with Louis Pasteur, Nocard joined Pasteur's laboratory in 1880. Nocard attended the famous experiments on the vaccination of sheep against anthrax in Pouilly-le-Fort. In 1883, a cholera epidemic struck Egypt. It threatened to spread to Europe, in consequence of the traffic generated by the new Suez Canal, which commenced operating in 1869. The Canal was built by the French and initially owned by the Egyptians and the French but the British, who took control in 1882, would have to manage this first epidemic since the germ theory acquired scientific credibility as the cause of this kind of epidemic. The aetiology and epidemiology issues were more geopolitical than medical for the British, with the risk of calling into question the economic viability of the Canal. In the event, it was the increased traffic through the Canal that brought pathogenic germs from India, a major British possession. Britain, France and Germany each sent teams to investigate the origin of this cholera outbreak.

The British were bent on demonstrating that the cholera was not caused by boats coming from India but due to poor local hygiene conditions. They proposed that the problem would soon be resolved, thanks to their governance. However, at that time, only France and Prussia had teams of bacteriologists capable of documenting the aetiology and epidemiology of the epidemic. Pasteur sent Roux to Egypt. Roux was supported by Nocard, his veterinarian. Nine days later Koch arrived, heading a German government commission (26). To justify the presence of Nocard, Pasteur wrote to the minister in charge of the project, to explain that it was impossible to directly study contagious diseases in humans and that his research programme necessarily included experiments on animals, thus requiring the presence of a veterinarian familiar with experimental infections (30). Unfortunately, the French delegation returned home quickly, following the death of one of its four members from cholera. It was Koch who then provided most of the scientific knowledge on the epidemic, including its Indian origin (26). This did not prevent British officials from challenging all Koch's explanations; his conclusions were allegedly "dangerous and unverifiable". The

ultimate goal of the British was to dismantle the Mediterranean quarantine system, with the intent of achieving unrestricted passage through the Suez Canal to India.

On his return from Egypt, Nocard established an annex to Pasteur's laboratory at the Alfort Veterinary School. Applying Pasteurian precepts, he was responsible for many scientific advances (30), involving the establishment of bridges between veterinary and human medicine. His research on the prevention of tuberculosis led to a famous quotation, displayed on Parisian transport, prohibiting spitting in the omnibuses and trams. Nocard demonstrated, with Chauveau, the identical nature of human tuberculosis and tuberculosis in domestic animals. Moreover, by studying the relationship between tuberculosis in humans and birds, he concluded that the agents of these diseases were two variants of the same species. In addition, he: participated in the development of diphtheria serotherapy; studied the bacteria responsible for mastitis in cows; and discovered mycoplasmas by identifying the cause of bovine pleuropneumonia (30). He weaponised tuberculin and mallein major in the fight against bovine tuberculosis and equine glanders, two bacterial diseases transmissible to humans. A bacterial genus was named Nocardia in his honour. He died prematurely in 1903 at the age of 53, yet he contributed to a major medical breakthrough after his death, by supplying his student, Camille Guérin, with the original strain of bovine mycobacteria used in the BCG vaccine.

Schüzt was a veterinarian who supported Koch and Friedrich Löffler, in the same manner that Nocard supported Pasteur and Emile Roux. Schütz (1839-1920) was born in Berlin. He became a licensed veterinarian in 1860. From 1860 to 1864 he studied human medicine. In 1876 he was appointed as professor of pathological anatomy,bacteriology and pathology at the Tierarzneischule Berlin, and served three terms as its “Rektor” (Dean). In an obituary account, Schütz was described as the “founder of the pathological veterinary anatomy” (31). Until 1868 he was an assistant and mentee of Rudolf Virchow. His contributions to bacteriology were made in association with Robert Koch, working in the “Kaiserliches Gesundheitsamt”, adjacent to the Tierarzneischule Berlin. He learned bacteriological techniques from Friedrich Löffler, the first assistant of Robert Koch. In 1882, they cultured Burkholderia mallei, the pathogen causing snot (glanders) (32). Later he established diagnostic tools for the detection of snot. This was applied in World War One in horses of the German army. Together with Robert Koch, he discovered the swine red rash bacillus and described its occurrence also in in horses (33). They also described the differences between the tuberculosis pathogen in cattle and humans (34). This fuelled the erroneous view of Koch that human bacilli could not infect cattle and vice versa. Koch’s

assertion caused consternation in Britain, where royal commissions had declared ingestion of tuberculous matter in food to be dangerous, and milk from cows with tuberculous udders had recently been banned for human consumption (26). In 1887, Schutz isolated *Streptococcus equi* as the causative agent of strangles. He retained close ties to the Tierarzneischule Berlin and was actively involved in research until his death in 1920 (35), (36).

References

1. Auyang SY. Reality and politics in the war on infectious diseases [Internet]. Available from: http://www.creatingtechnology.org/biomed/germs.htm
2. Anonymous. Joseph Lister. In Wikipedia; Available from: https://en.wikipedia.org/wiki/Joseph_Lister
3. Anonymous. Pasteur, louis. In: Encyclopedia.com [Internet]. Available from: https://www.encyclopedia.com/people/science-and-technology/chemistry-biographies/louis-pasteur
4. Chaloner EJ. Amputation at the London hospital 1852-1857. Journal of the royal society of medicine. 2001;(94):409–12.
5. Brand RA. Biographical Sketch: Baron Joseph Lister, FRCS, 1827-1912. Clinical Orthopaedics & Related Research. 2010 Aug;468(8):2009–11.
6. Anonymous. Today in science history: Lord Joseph Lister [Internet]. 1864. Available from: https://todayinsci.com/L/Lister_Joseph/ListerJoseph-Quotations.htm
7. Lister BJ. The Classic: On the Antiseptic Principle in the Practice of Surgery. Clinical Orthopaedics & Related Research. 2010 Aug;468(8):2012–6.
8. Lister J. On the Antiseptic Principle in the Practice of Surgery. BMJ. 1867 Sep 21;2(351):246–8.
9. Tröhler U. Statistics and the British controversy about the effects of Joseph Lister's system of antisepsis for surgery, 1867–1890. J R Soc Med. 2015 Jul;108(7):280–7.
10. Anonymous. Lister, Joseph, Baron Lister of Lyme Regis (1827 - 1912) [Internet]. Royal college of surgeons of england. Available from: https://livesonline.rcseng.ac.uk/client/en_GB/lives/search/detailnonmodal/ent:$002f$002fSD_ASSET$002f0$002fSD_ASSET:372684/one?qu=%22rcs%3A+E000500%22&rt=false%7C%7C%7CIDENTIFIER%7C%7C%7CResource+Identifier
11. Vallery-Radot R. The life of Pasteur [Internet]. The Project Gutenberg EBook of The life of Pasteur. 2019. Available from: https://www.gutenberg.org/files/60956/60956-h/60956-h.htm

12. Koch R. Die Ätiologie der Milzbrand-Krankheit, begründet auf die Entwicklungsgeschichte des Bacillus Anthracis. 2010 Mar 24 [cited 2021 Jun 28]; Available from: https://edoc.rki.de/handle/176904/5139
13. Centers for Disease Control and Prevention. History of Anthrax [Internet]. Available from: https://www.cdc.gov/anthrax/basics/anthrax-history.html
14. Koch R. Verfahren zur Untersuchung, zum Konservieren und Photographieren der Bakterien. 2010 Mar 24 [cited 2021 Jun 28]; Available from: https://edoc.rki.de/handle/176904/5140
15. Toussaint H. De l'immunite´ acquise a` la suite d'inoculations pre´ventives,. C R Acad Sci Paris,. 1880;(91):135–7.
16. Lister Joseph. REMARKS MICRO-ORGANISMS: THEIR RELATION TO DISEASE. The british Medical Journal. 1880;sept 4:363–5.
17. Tansey EM. "The Queen has been dreadfully shocked": aspects of teaching experimental physiology using animals in Britain, 1876-1986. Am J Physiol. 1998 Jun;274(6 Pt 2):S18-33.
18. Lister Joseph. An Address ON THE TREATMENT OF WOUNDS. The Lancet. 1881;(nov 19):863–6.
19. Anonyme. ELIE METCHNIKOFF (1845-1916) [Internet]. Institut pasteur; notre histoire. Available from: https://www.pasteur.fr/fr/institut-pasteur/elie-metchnikoff-prix-nobel-1908
20. Sandle T. History and development of microbiological culture media. Journal Institute of Science and Technology. 2011;10–4.
21. Blevins SM, Bronze MS. Robert Koch and the 'golden age' of bacteriology. International Journal of Infectious Diseases. 2010 Sep;14(9):e744–51.
22. Lagier J-C, Edouard S, Pagnier I, Mediannikov O, Drancourt M, Raoult D. Current and Past Strategies for Bacterial Culture in Clinical Microbiology. Clin Microbiol Rev. 2015 Jan;28(1):208–36.
23. Pasteur L, Chamberland C, Roux E. Summary report of the experiments conducted at Pouilly-le-Fort, near Melun, on the anthrax vaccination, 1881. Yale J Biol Med. 2002 Feb;75(1):59–62.
24. Loir A. A l'ombre de Pasteur : Souvenirs personnels. Le mouvement sanitaire. Paris; 1938. 171 p.
25. Koch R. ANTHRAX VACCINATION. KOCH'S REPLY TO PASTEUR. The Lancet. 1883;March 17:472–3.
26. Anonymous. Koch, Heinrich Hermann Robert. In: Encyclopedia.com [Internet]. Available from: https://www.encyclopedia.com/people/medicine/medicine-biographies/robert-koch

27. Perrot A, Schwartz M. Pasteur et Koch: Un duel de géants dans le monde des microbes. Odile Jacob sciences. Paris; 2014.
28. Saleem A, Azher M. The Next Pandemic - Tuberculosis: The Oldest Disease of Mankind Rising One More Time. British Journal of Medical Practionners. 2013;6(2):615-.
29. Sakula A. BCG: who were Calmette and Guérin? Thorax. 1983 Nov;38(11):806–12.
30. Orth G, Guenet J-L. L'ŒUVRE SCIENTIFIQUE D'EDMOND NOCARD (1850-1903). Bull.soc.fr.hist.méd.sci.vét. 2003;2(2):100–10.
31. Schwartz B. OBITUARY. Science. 1938 May 20;87(2264):451–3.
32. Loffler F, Schutz W. Einige Bemerkungen betreffend die Entdeckung des Rotzbazillus, D. Med. W. 1883, Nr. 14, page 197. Deutsche Medizinische Wochenschrift. 1883;(14):197.
33. Schutz W. Die influenza erysipelatosa (Rotlaufseuche) der Pferde. Archiv für wissenschaftliche und praktische Tierheilkunde. Archiv für wissenschaftliche und praktische Tierheilkunde. 1882;
34. Koch R, Schutz W. Bericht über die menschliche Tuberkulose und die Rindertuberkulose (Perlsucht). Archiv für wissenschaftliche und praktische Tierheilkunde. 1902;169.
35. Schmaltz R. Der letzte der Pioniere [J.W. Schütz]. Berliner tierärztliche Wochenschrift. 1920;36(52):624–8.
36. Wernicke RK. H. Von der Zootomie zur neuzeitlichen Pferdeheilkunde. Entwicklung der Tiermedizin in Berlin-Mitte Pferdeheilkunde. 2005;21:327–40.
